# Human Serum Albumin Labelled with Sterically-Hindered Nitroxides as Potential MRI Contrast Agents

**DOI:** 10.3390/molecules25071709

**Published:** 2020-04-08

**Authors:** Sergey Dobrynin, Sergei Kutseikin, Denis Morozov, Olesya Krumkacheva, Anna Spitsyna, Yurii Gatilov, Vladimir Silnikov, Goran Angelovski, Michael K. Bowman, Igor Kirilyuk, Alexey Chubarov

**Affiliations:** 1 N.N. Vorozhtsov Institute of Organic Chemistry, SB RAS 630090 Novosibirsk, Russia; s.a.dobrynin@gmail.com (S.D.); m_falcon@nioch.nsc.ru (D.M.); a.spitsyna@alumni.nsu.ru (A.S.); gatilov@nioch.nsc.ru (Y.G.); 2 Institute of Chemical Biology and Fundamental Medicine, SB RAS 630090 Novosibirsk, Russia; kutseykin@gmail.com (S.K.); silnik@niboch.nsc.ru (V.S.); 3 International Tomography Center, SB RAS 630090 Novosibirsk, Russia; olesya@tomo.nsc.ru; 4 Max Planck Institute for Biological Cybernetics, 72076 Tubingen, Germany; goran.angelovski@tuebingen.mpg.de; 5 Department of Chemistry & Biochemistry, University of Alabama, Tuscaloosa, AL 35487, USA

**Keywords:** nitroxide radicals, spin probes, organic radical contrast agents, human serum albumin, homocysteine thiolactone, magnetic resonance imaging

## Abstract

Four albumin-nitroxide conjugates were prepared and tested as metal-free organic radical contrast agents (ORCAs) for magnetic resonance imaging (MRI). Each human serum albumin (HSA) carrier bears multiple nitroxides conjugated via homocysteine thiolactones. These molecular conjugates retain important physical and biological properties of their HSA component, and the resistance of their nitroxide groups to bioreduction was retained or enhanced. The relaxivities are similar for these four conjugates and are much greater than those of their individual components: the HSA or the small nitroxide molecules. This new family of conjugates has excellent prospects for optimization as ORCAs.

## 1. Introduction

Magnetic resonance imaging (MRI) is a powerful non-invasive technique for clinical diagnosis. The specificity of MRI is sometimes insufficient and can be improved by enhancing the spin relaxation of water protons by the addition of a contrast agent. The spin relaxation of protons is characterized by two parameters, *T_1_* and *T_2_*, called the spin-lattice and spin-spin relaxation times, respectively. Depending on their effect on *T_1_* and *T_2_*, contrast agents fall into two classes that afford positive and negative contrast images, respectively [1,2,3,4]. The most efficient of the currently-used *T_1_* contrast agents are paramagnetic transition metal chelates of gadolinium and manganese. However, the low stability of some chelates is a potential challenge due to the release of potentially toxic metal ions from the chelates [1,5,6,7,8,9]. Consequently, the European Medicines Agency recently recommended restricting the use of less stable linear Gd(III) complexes and using macrocyclic Gd(III) agents at the lowest possible dose [6,7,8]. Magnetite/maghemite combinations approved for clinical use, as *T_2_* MRI contrast agents have little or no cytotoxicity but may have harmful cellular effects, including oxidative stress, reactive oxygen species (ROS) generation, mitochondrial membrane dysfunction, DNA damage, mutagenicity, changes in gene expression, etc. [10,11,12] Therefore, there is extensive interest in the production and investigation of “metal-free” MRI contrast agents. 

Heteronuclear MRI (e.g., ^19^F MRI, [13,14] imaging ^19^F nuclei administered to the patient rather than the ubiquitous protons) is a complementary metal-free approach with great potential, good sensitivity and essentially no background signals in tissue. However, such techniques require high (mM) concentrations of the contrast agent and use imaging protocols that are not currently common in the clinic [4,15]. Therefore, there is an impetus to develop organic radical contrast agents (ORCA) [4,15,16,17,18,19,20,21,22,23], with nitroxide stable radicals conjugated to macromolecules, which are compatible with standard imaging protocols as alternatives to metal-containing contrast agents. 

Organic radical contrast agents (ORCAs) have the same MRI mechanism as metal-based contrast agents and can use similar imaging protocols. One big advantage is that they should be well tolerated by patients if assembled from highly biocompatible components. ORCAs do face potential hurdles. First, the most common nitroxides undergo rapid reduction reactions in vivo to diamagnetic compounds [24], eliminating their contrast-enhancing abilities. However, there are sterically-hindered nitroxides having much slower rates of reduction; some were recently used for intracellular measurements and provided useful lifetimes and biocompatibility [25]. Biological oxidation generally is not a concern because nitroxides are much more resistant to oxidation [26,27,28]. In fact, oxidation can restore some bioreduced nitroxides back into active nitroxides. A second difficulty is that each nitroxide group has only one unpaired electron, resulting in low relaxivity per nitroxide group and poor efficacy compared to metal ion contrast agents which have several unpaired electrons. But polyradical ORCAs can have sufficient relaxivity per molecule by conjugating multiple free radicals to a macromolecular or supramolecular nanoparticle carrier [15,16,17,19,22,23,29,30,31]. 

Here we develop a class of macromolecular constructs from nitroxides conjugated to a human carrier protein as potential ORCAs. These new conjugates retain important properties of their components, such as high biocompatibility and paramagnetism, while other properties, notably relaxivity, are significantly enhanced over those of the components. Understanding how properties of these conjugates derive from their component molecules can enable their optimization for use as metal-free ORCAs. 

The carrier needs to be highly biocompatible and readily disposable by the patient. We can minimize harmful impacts by using a carrier that humans readily process and eliminate, in order to minimize the mass of synthetic or xenobiotic material in the ORCA. We think that the carrier should have several qualities: (1) high abundance in the patient so that the ORCA adds a negligible load for disposal to what is naturally present; (2) a concentration that is not tightly regulated so the ORCA’s increment is well tolerated; (3) high solubility and wide distribution in bodily fluids; and (4) high biocompatibility and robustness toward chemical modification. 

These desired qualities make human serum albumin (HSA) a very attractive carrier for an ORCA. HSA is the most abundant protein in blood plasma. One of its physiological roles is to carry small polar and non-polar molecules, often several at a time, and it has attracted attention as a carrier for therapy and diagnostics [32]. Its typical concentration in human serum is high, 34–54 g/L or 0.5–0.8 mM, and it carries a wide range of natural post-translational modifications, showing robust structure and properties in the face of chemical modification.

**Chart 1:**
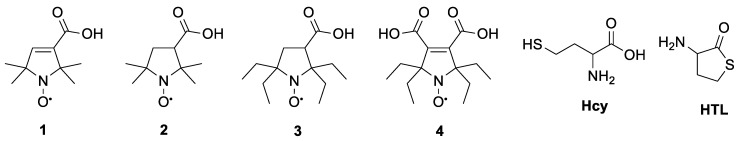
Nitroxide carboxylic acids **1**–**4**, homocysteine (Hcy) and homocysteine thiolactone (HTL) molecules.

The cysteine amino acid residue Cys34 of albumin is often used for site-specific chemical conjugation of drugs or probes [33,34,35]. However, HSA harbors 59 lysine residues, which also can be used for covalent conjugation [36,37,38]. The high Lys abundance makes the modification of a single, specific site a technical challenge, but offers an attractive route to albumin-based ORCAs that use many Lys to load, carry and deliver multiple nitroxide groups. We have suggested using a natural posttranslational modification of HSA to label Lys [39,40]. Homocysteine thiolactone (HTL) (Chart 1) adducts HSA via an irreversible acylation of the *ε*-amino group of Lys residues, called *N*-homocysteinylation. The predominant site for *N*-homocysteinylation of HSA is Lys-525 in vitro and in vivo, but four other homocysteinylation products (adducted at Lys-212, 137, 12 and 4) are seen in vivo [41,42], and two additional Lys are modified in vitro, for a total of seven (at Lys-525, 212, 205, 159, 137, 12 and 4) [41,42,43]. Homocysteinylation is not random and the sites and extent of modification are under some control in the construction of new conjugate molecules.

HTL can be chemically *N*-acylated by a nitroxide group to produce a reagent for making *N*-Hcy-acylated *N*-homocysteinylated albumin. The wide range of known nitroxide free radicals includes bioreduction-resistant nitroxide groups that can be a basis for combining the attractive properties of HSA, such as penetrating blood-vessel barriers, passing through the blood-brain barrier and high biocompatibility, with metal-free nitroxides that resist bioreduction.

In the present work, we test the validity of this ORCA design strategy by preparing a set of spin labels via *N*-acylation of HTL with four nitroxide carboxylic acids **1**–**4** (Chart 1) and testing the suitability of their HSA-nitroxide conjugates (HSA-NIT) as metal-free ORCAs for MRI. The conjugates were characterized by electron paramagnetic resonance spectroscopy (EPR), matrix assisted laser desorption/ionization time of flight mass spectrometry (MALDI ToF MS) and circular dichroism (CD) spectroscopy. Nitroxide reduction rates in these biomaterials were measured; the relaxivities *r_1_* and *r_2_* were estimated; and ^1^H-MRI phantom images were obtained. Initial tests of biocompatibility checked for the formation of potentially toxic aggregates using sodium dodecyl sulfate polyacrylamide gel electrophoresis (SDS-PAGE) and for cytotoxicity of the conjugates using standard cellular assays [44,45]. The results from these first four conjugates show great promise for homocysteinylation of HSA in the construction of ORCAs, while the wide variety of known nitroxide compounds provide strong potential for optimizing the performance of this new family of ORCAs.

## 2. Results

### 2.1. Synthesis of N-Substituted Homocysteine Thiolactone Derivatives

**Figure 1:**
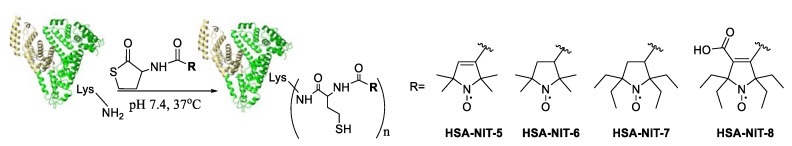
Synthetic route to obtain HSA-nitroxide conjugates (HSA-NIT). HSA is shown schematically as a heart-like structure of helical ribbons.

Homocysteine thiolactone (HTL) is a natural cyclic product from the intramolecular acylation of homocysteine (Hcy) (Chart 1). Some aminoacyl-tRNA synthetases form HTL in their editing and proofreading reactions in order to prevent translational incorporation of Hcy into proteins [46]. HTL easily reacts with nucleophilic and electrophilic reagents at its activated carboxyl and amino groups, respectively. Reaction of HTL with the nucleophilic terminal amine of Lys can be performed in aqueous media, opening the five-membered ring of HTL and producing a stable amide bond (Figure 1).

**Scheme 1:**
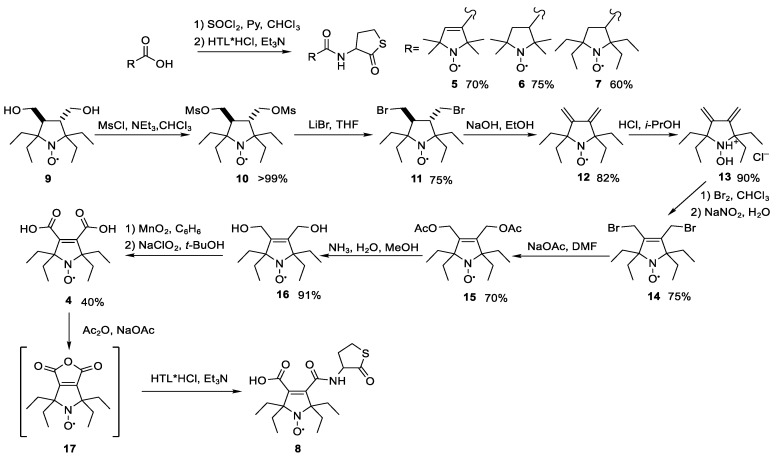
Synthesis of *N*-substituted homocysteine thiolactone derivatives **5**–**8**.

Electrophilic reactions to attach a nitroxide to the HTL amino group face the difficulty that the hydrochloride of HTL is chemically stable in bulk [46]. Attempts to obtain the free base for reaction results in the self-condensation of two HTL molecules [47,48]. The intrinsic instability of the HTL free base requires an electrophile with enhanced reactivity for efficient reaction with the amino group; examples include acid halides, activated carboxylic acids and anhydrides [39,49,50]. Therefore, commercially-available nitroxide carboxylic acids **1** and **2**, as well as the sterically-shielded, reduction-resistant nitroxide **3** [51], were first converted in situ into chloroanhydrides with thionyl chloride and then used to construct ORCAs. A racemic mixture of D and L isomers of HTL hydrochloride was then added as a powder and the solution was carefully basified again (Scheme 1). The reaction with **1** afforded the racemic spin-labeled HTL **5** as a yellow crystalline solid. The IR spectrum showed strong bands of thiolactone and amide carbonyl vibrations at 1706 and 1664 cm^−1^, respectively; pyrroline C=C band at 1620 cm^−1^; and multiple broad bands of hydrogen-bonded NH vibrations at 3361, 3321 and 3054 cm^−1^ (See Supplementary Materials for full synthetic and analytical details). High-resolution mass spectral and element analysis data support the assigned structure. For further confirmation, the nitroxide was converted into its corresponding methoxyamine [52] and the ^1^H NMR spectrum was recorded. Acylation of HTL with **2** and **3** gave mixtures of diastereomers, **6a, b** and **7a, b**. The isomers **6a** and **6b** were partly separated using chromatography and the structure of **6a** was confirmed (Figure S11.1) by single-crystal X-ray analysis (all calculations were performed using SHELXTL-2018/3 [53]). The isomers **7a, b** could not be separated, so their mixture was characterized.

The sterically-shielded nitroxide **7** (as a diastereomeric mixture) is hydrophobic and poorly soluble in water, which complicates its use for labeling proteins in water solutions, so the more hydrophilic spin label **8** was prepared from the recently-synthesized nitroxide **9 [54]**. Conversion of **9** into **4** (Scheme 1) followed the strategy for its 2,2,5,5-tetramethyl analog [55,56]. Mesylation afforded **10** in nearly quantitative yield. The reaction of **10** with LiBr appeared much smoother than the similar reaction with NaI and the resulting dibromide **11** required somewhat longer time for dehydrobromination than did the dehydrohalogenation of 3,4-bis-iodomethyl-2,2,5,5-tetraethyl-pyrrolidine-1-oxyl [56]. Reduction of the resulting diene **12** with the 2-propanol–hydrogen chloride system afforded **13**, which was brominated as described for its 2,2,5,5-tetramethyl analog [56]. Nucleophilic substitution with NaOAc, aminolysis and two-step oxidation afforded **4**. The spectral parameters of the new compounds are similar to those of their tetramethyl analogs [55,56]. The structures of **16** and **13** were confirmed by X-ray analysis data (Figure S11.1). The acid **4** was heated with acetic anhydride and sodium acetate to form the cyclic anhydride **17** (cf. [55]), which was immediately used for HTL acylation without further purification. The spectral parameters of **8** were similar to those of **5**; the structure was confirmed with elemental analysis and high-resolution mass spectra.

### 2.2. Synthesis and Characterization of HSA-Nitroxide Conjugates

*N*-Homocysteinylation of HSA by HTL-nitroxide derivatives **5–8** was done under physiological-like conditions (PBS, 37 °C, pH 7.4) (Figure 1 and Supplementary Materials). Unreacted reagent and other low MW compounds were removed by centrifugal filtration using Centricon concentrators. The yields of HSA-NIT conjugates were 95%. For comparison, *N*-homocysteinylated HSA was synthesized. The reaction conditions were optimized with respect to DMSO content, HSA concentration and the excess of HTL or HTL derivatives. The incorporation of Hcy-nitroxide residues into HSA was proven by MALDI-ToF, EPR and Ellman’s assay for free SH-groups [57], with unmodified HSA as control.

A new SH-group is introduced in the resulting HSA by each *N*-homocysteinylation reaction. The albumin SH-group content and measured extent of reaction increased with increasing molar ratios of acylating reagent (HTL or HTL derivative) to protein (Figure S3.1). The extent of HSA modification does not increase significantly with greater than a 30-fold excess of HTL derivatives, which can be explained as the acylation of the main albumin *N*-homocysteinylation sites. 

The extent of modification derived from Ellman’s test was much lower than from MALDI ToF or EPR (Tables S1 and S2), an artifact of the use of DMSO. Despite its wide use as a safe solvent for pharmacological substances and for biopolymer modification reactions, DMSO can cause protein conformational changes and oligomerization [58,59]. DMSO is sometimes used as a mild oxidizing agent for SH groups [60,61]. Therefore, the lowest possible dose of DMSO was used to minimize its adverse effects.

*N*-homocysteinylation, like any protein modification, might cause protein damage, loss of function, conformational changes and precipitation; or might change susceptibility to oxidation, proteolysis and oligomerization [46,48,62,63]. Gel electrophoresis (SDS-PAGE) was used to check the HSA-NIT for any changes in mobility from that of HSA (Table 1) that could indicate aggregation or protein damage. Bands corresponding to monomeric protein (MW = 66.5 kDa) and its dimer (MW ~ 130 kDa) were observed in all samples, including the starting HSA, but the HSA-NIT conjugates showed no significant changes from the starting, natural HSA (Figure S3.3).

Changes in the secondary structure of the protein were examined by deconvolution of CD spectra to determine the *α*-helix and *β*-sheet content (Table 1) based on CCA+ software [64,65]. The secondary structure of the HSA remained intact: *α*-helical content of the HSA-NIT conjugates decreased slightly from 55% to 46%–48%, while the *β*-sheet content was almost unchanged. *N*-homocysteinylation of HSA by HTL produced a slight increase in *β*-sheets (Hcy-HSA, Table 1) [47,66]. The positive charge of the Hcy amino group and its interaction with a protein side chain cause these slight conformational adjustments [47], but CD and SDS-PAGE show that the structural integrity of the HSA was preserved in all four conjugates.

### 2.3. Identification of N-Homocysteinylation Sites in HSA-NIT Conjugates 

The HSA sites modified by the HTL derivatives were identified using trypsin digestion with MALDI-ToF peptide mapping. Each HSA-NIT conjugate is a heterogeneous mixture having Hcy-nitroxide residues attached at different lysine residues (Table S4). The major modified sites coincide with the *N*-homocysteinylation sites of HSA in vitro and in vivo [41,42,43] (Table 2). We were able to map sites over a large coverage of the HSA. Surprisingly, many additional Lys residues were modified by the *N*-substituted HTL derivatives: Lys-573/564, 564, 560, 519, 475, 466, 444, 439/436, 436, 432, 413, 351, 323/317, 274, 225, 199, 195, 190, 181, 162, 93, 73 and 64 (Table S4). *N*-homocysteinylation by HTL-nitroxide derivatives can occur at many previously unreported sites and offers the potential for ORCAs with very high loadings, or with additional diagnostic or therapeutic groups. 

Various factors, including protein conformation, nucleophilicity, local charges and p*K*_a_, determine the reactivity of potential acylation sites, and can help explain the sites of modification. The p*K*_a_ value and the fractional accessible surface area (ASA) were estimated for each Lys side chain [67,68,69]. Lys residues with low p*K*_a_ values should be unprotonated, and hence more active to nucleophilic substitution. Side chains with high ASA values are more accessible to modifying reagents. For example, Lys-525 has the lowest calculated p*K*_a_ among the residues usually *N*-homocysteinylated, reflecting this trend (Table S5). Consequently, the pattern of Lys modification in HSA-NIT broadly resembles that of natural HSA post-translational modification which helps preserve biocompatibility.

**Figure 2:**
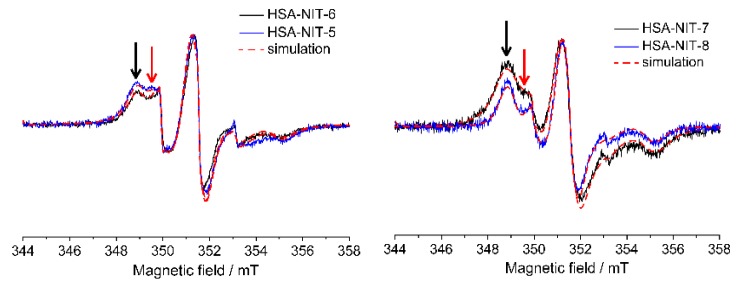
X-band CW EPR spectrum of spin-labelled conjugates at 300 K. Black and blue lines with noise are experimental spectra. The experimental spectra were simulated using EasySpin software [71,72]. Red lines are simulated spectra with parameters listed in Table S3. Red arrow—«fast», black—«slow» motion component.

Attachment of the nitroxide groups to HSA was verified by room-temperature continuous-wave (CW) EPR spectra (Figure 2). Binding of a nitroxide to any protein significantly increases its rotation correlation time to >1.0 ns, producing pronounced changes in its CW EPR spectrum [70]. Spectral modeling indicates that 99% of the nitroxides are attached to the HSA and exhibit slowed rotation with τ_c_ >1.0 ns (Table S3). The remaining <1% are trace amounts of incompletely washed-out free **5**–**8**, freely tumbling in solution and unaffected by the protein (τ_c_~0.6 ns).

The EPR spectra from nitroxides at each modification site overlap, preventing characterization of individual modification sites by EPR. However, the partially-immobilized labels can be classified by motional correlation time (τ_c_)—2–3 ns («fast») or 10–14 ns («slow»)—and attributed to labels located outside and inside pockets of the protein, respectively (Table 3 and Table S3). Most labels are in the «slow» class whose long *τ_c_* reveals a rather confining environment [15,70]. This characterization is supported by their nitrogen hyperfine constants (HFC): the «slow» labels have a smaller HFC resulting from their less polar environment inside the protein, while the «fast» labels are less hindered in the polar surroundings at the exposed HSA surface [65]. The strongly immobilized «slow» labels comprise the majority in all samples, especially those with tetraethyl substituted radicals.

### 2.4. Reduction Rates of HSA-NIT Conjugates

Bioreduction of nitroxides to diamagnetic hydroxylamines, which is typical under physiological conditions [26,27,28,74], negatively affects ORCA relaxivity and complicates their use as MRI contrast agents. Bio-oxidation is rare and not a problem [28,75]. We evaluated the reduction rates of nitroxide groups in the conjugates and in **1**–**4** by ascorbate, the reducing agent standardly used to estimate the redox properties of nitroxides [21,29,54]. Reduction rates were measured under pseudo-first-order conditions using a 400-fold excess of ascorbate (≈100–150 per nitroxide unit) and a 200-fold excess of glutathione per albumin molecule in pH 7.4 PBS. Second-order rate constants for reduction were obtained: (1) from the initial decay of the low-field EPR peak amplitude (labelled init.) with calibration by the double integral of the EPR spectrum; and (2) from the change of the ^1^H NMR *r_1_*-relaxation rate with time. As seen in Table 3, the *r_1_*-relaxation measurement is suitable only for the more stable nitroxides because it requires more time than is available with rapidly-decaying nitroxides. 

The nitroxide environment in the HSA-NIT conjugates can alter the rate of nitroxide reduction [15]. For example, reduction of **1** was significantly slower in its HSA-NIT-5 conjugate (*k* = 0.096 M^−1^s^−1^) than in a free solution of **1** (*k* = 0.22–0.58 M^−1^s^−1^). In contrast, the reduction of radical **3** seems unaffected by environment whether it is conjugated in HSA-NIT-7 or free in solution (Table 3). The reduction-resistance of nitroxides **3** and **4** can be preserved in HSA-conjugate ORCAs to enable MRI measurements over longer time scales.

We found above (Table 2, Table S4, and Section 5 of Supplemental Materials) that chemical reaction of HSA with HTL derivatives depends on the accessibility of the site. The same effect is seen in the reduction of nitroxides on the outside and inside of virus particle-based ORCAs, and can be expected in the HSA-NIT. Spectral simulations, made throughout the reduction, determined the amount of the «slow» and «fast» motional components. Those values were used to determine reduction rates of the nitroxides belonging to each motional component (Table 3). The reduction rate constants for the «slow» component are 2–5 fold slower than for the «fast» component. This difference is presumably because the protein provides reducing agents less access to the «slow» component. This effect provides a possible strategy to produce even more stable conjugates. 

The decay rates obtained from the initial decay of all the nitroxides (init.) agree well with the rates from the ^1^H *r_1_* measurements for the sterically-hindered HSA-NIT-7 and HSA-NIT-8. This suggests that nitroxides in both motional components contribute similarly to the relaxivity. There is little correlation between the EPR-derived reduction rates and those from the *r_1_* measurements for the rapidly reduced conjugates, which is likely an artifact from making a lengthy *r_1_* measurement on a rapidly-changing sample.

A slow reduction rate is vital for a nitroxide-based ORCA to provide consistent relaxivity for the MRI measurement. Thus, it is significant that under similar conditions, HSA-NIT-7 and HSA-NIT-8, with *k* = 0.0018 ± 0.0002 M^−1^s^−1^ and 0.0022 ± 0.0002 M^−1^s^−1^, respectively, are reduced more than an order of magnitude slower than the nitroxides on a dendrimer-based carrier proposed as a metal-free ORCA for MRI with *k* = 0.0376 M^−1^s^−1^ [15]. 

### 2.5. Cytotoxicity of HSA-NIT Conjugates

**Figure 3:**
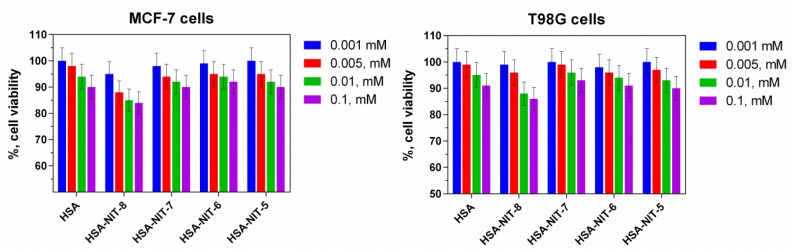
Effects of HSA-NIT conjugates on the viabilities of MCF-7 and T98G cell lines. Cell viability was normalized using cells treated with PBS buffer as a 100% viability control. There is no significant difference between HSA and any of the conjugates.

The viability of cells in the presence of the HSA-NIT conjugates was investigated by the widely-applied 3-(4,5-dimethylthiazol-2-yl)-2,5-diphenyltetrazolium bromide (MTT) test using breast cancer MCF-7 and human glioblastoma T98G cells [44,45]. During exponential growth phase, cell cultures were treated for 72 h with amounts of the HSA conjugates in the range expected for ORCA MRI applications (see discussion below). The HSA–NIT conjugates show no significant difference from native HSA (Figure 3).

### 2.6. Characterization of HSA-NIT Magnetic Properties

The ^1^H water relaxivities *r_1_* and *r_2_* of the HSA-NIT conjugates in PBS were measured using a 7 T Bruker Avance III 300 MHz spectrometer at 25 °C (Table 4). The plots of the ^1^H relaxation rate of water (1/*T_1_*) versus the nitroxide concentration were linear for all agents, indicating the absence of aggregation (Figure S7.1). The per-nitroxide *r_1_* values ranged from 0.33 to 0.51 mM^−1^s^−1^ (Table 4), making them comparable to those reported in various nitroxide-bound polymers and dendrimers [15,29] and higher than for potential low-molecular-weight, nitroxide-based MRI probes at 7 T [20,21]. Moreover, per-nitroxide *r_2_* values ranged from 4.7 to 7.2 mM^−1^s^−1^, much greater than what is typical for individual nitroxides in solution; e.g., **2** has *r_1_* and *r_2_* of 0.15 and 0.17 mM^−1^s^−1^, respectively [76]. 

HSA-NIT-6 and HSA-NIT-7 contain nitroxides with similar ring structures and have good relaxivities, but have reduction rates differing by about half an order of magnitude; so we compared their relaxivities at the physiological temperature of 37 °C (Table 4). The *r_1_* and *r_2_* values decreased similarly as expected, with the *r_2_/r_1_* ratios virtually unchanged. The molecular structure features of the nitroxide group that control reduction rates do not seem to significantly affect relaxivity or its temperature dependence.

In fact, the *r_2_* per nitroxide group (4.3–5.3 mM^−1^s^−1^) for both conjugates, at 37 °C and 7 T, match or exceed those reported for other ORCAs (0.17–4.7 mM^−1^s^−1^) [23]. This was achieved at a relatively light loading of 3.9 nitroxides per HSA (Table 4). However, HTL spin labels can be possibly attached to at least 23 Lys residues (Table S4), providing scope for increasing, as much as 5-fold, the loading and the relaxivity per HSA.

**Figure 4:**
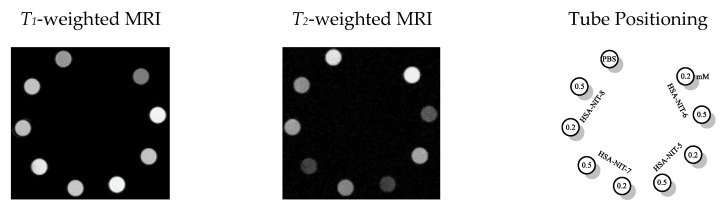
MRI phantoms of HSA-NIT conjugates imaged in a 7 T BioSpec 70/30 USR magnet at RT. *T_1_* and *T_2_*-weighted MRI phantoms at HSA-NIT concentrations (0.2 mM and 0.5 mM). Acquisition parameters are provided in ESI.

*T_1_*- and *T_2_*-weighted MRIs on tube phantoms of the HSA-NIT biomaterials in PBS solutions show excellent contrast between samples with 0.0, 0.2 and 0.5 mM concentrations at 7 T and RT (Figure 4). There is no significant difference in contrast efficiency between the different types of nitroxides. The contrast differences among samples correlate principally with their level of loading with nitroxide. 

Positive and negative contrast enhancements are clearly observed in *T_1_* and *T_2_* images, respectively. The *r_2_*/*r_1_* values of 10.4–14.3 obtained in a 7 T magnetic field suggest that at high field strengths the conjugates may work most effectively as *T_2_* contrast agents [15,19]. The *r_2_*/*r_1_* values per nitroxide exceed those of most other nitroxide-based ORCAs [15,20,23] and the *r_1_* relaxivities are similar to values at 7 T for other ORCAs.

The MRI performance of HSA-NITs is quite encouraging because the four conjugates characterized here already show good relaxivity and indicate a scope for substantial performance increases for this new class of ORCAs. The large number of known nitroxide molecules provides a range of chemical and physical properties for optimizing an HSA-based ORCA. The high solubility of HSA and biocompatibility of HSA-NIT ORCAs can also bring advantages. HSA readily crosses blood-vessel and blood-brain barriers and can accumulate in tumors; hence HSA conjugates can be injected remotely from sensitive organs/tissues and be delivered by the circulation. The properties of HSA are preserved when conjugated with HTL or its derivatives [40,47,77] (and see above), and could be exploited with ORCAs to specifically enhance contrast for tumors. HSA-based constructs often bind to albondin on the endothelium and SPARC in the tumor interstitium, thereby accumulating in and highlighting tumors, including brain tumors (glioma) [32,40,47,77,78]. Finally, the lifetime of HSA in the body is about 25 days with a normal turnover of more than 10 g per day [79], readily accommodating the HSA added by a conjugate optimized for use as an ORCA.

## 3. Conclusions

A new family of nitroxide-bearing conjugates (HSA-NIT) was constructed by the attachment of four different HTL-based spin labels to human serum albumin. These conjugates retain important physical and biological properties of their HSA component, and the chemical resistance of their nitroxide groups was retained or enhanced. The relaxivities of these four conjugates are similar and are much greater than those of their components: the HSA or the small nitroxide molecules. 

It is possible to homocysteinylate HSA at previously-undocumented Lys locations, providing the potential of much heavier loading of nitroxides for HSA-NIT conjugates with even greater relaxivities, potentially surpassing those of the best current ORCAs. The combination of the inherent biocompatibility and long blood half-life of albumin, in addition to the possibility for these albumin conjugates to accumulate in tumors and the excellent stability of their nitroxide radicals under reducing conditions, can provide promising contrast media for MRI diagnostics. Last but not least, these HSA-NIT conjugates are also an extensible basis for the development of theranostics that combine ORCAs with therapeutic payloads to achieve simultaneous drug delivery and tumor imaging.

## Figures and Tables

**Table 1:** HSA conjugate secondary structure from CD and aggregates from quantitative SDS–PAGE analysis.

		CD Spectral Analysis		SDS-PAGE Analysis	
HSA Type	n *	*α*-Helix, %	*β*-Sheet, %	Oligomer, %	Monomer, %
HSA	0	55.0	4.5	16	84
HSA-NIT-5	4.8	45.8	4.8	17	83
HSA-NIT-6	3.9	46.5	5.6	18	82
HSA-NIT-7	3.9	46.2	5.1	17	83
HSA-NIT-8	2.4	48.0	5.0	15	85
Hcy-has	3.3	44.0	8.0	41	59

* Hcy residues per HSA molecule.

**Table 2:** Lysines in *N*-Hcy-albumin conjugates modified by Hcy.

HSA Type	*N-Hcy site (Lys residues)* *						
	525	212	205	159	137	12	4
HSA-NIT-5	++	+	++	+	+	++	+
HSA-NIT-6	++	+	++	+	+	+	0
HSA-NIT-7	++	+	++	+	++	+	+
HSA-NIT-8	++	+	++	+	+	+	++
Hcy-HSA [41,42,43]	++	++	+	+	++	+	+
PFT-Hcy-HSA [40]	++	0	++	0	++	0	0

* 0: no peptides found modified at this site; + minor site: one or two peptides modified at this site; ++ major site: three or more peptides modified at this site.

**Table 3:** Second-order rate constants *, *k* (M^−1^s^−1^) for initial rates of reduction of nitroxides with ascorbate/glutathione solution in pH 7.4 at 25 °C.

Nitroxide Structure	Method					Nitroxide Structure	Method	
	EPR				*r_1_*-meas.		EPR	*r_1_*-meas.
	init.	«fast»	«slow»					
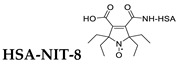	0.0010	0.0025		0.0006	0.0022 ± 0.0003	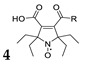	-	-
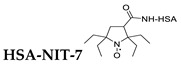	0.0017	0.005		0.0015	0.0018 ± 0.0003	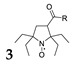	R=OH ~0.0013 [73] 0.002 [74]	R=OH 0.0023
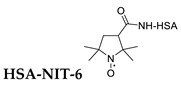	0.056	0.13		0.053	0.008 ± 0.001	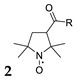	R=OH 0.063 [26,29] 0.097 [27] R=NH2 0.30 [27]	R=OH n.d.**
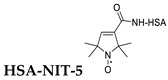	0.096	0.22		0.066	0.015 ± 0.003	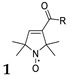	R=OH 0.22 [27] R=NH_2_ 0.58 [27]	R=OH n.d.**

* Kinetics of nitroxide reduction are summarized in Figure S6.1 and Figure S6.2; ** rapid reduction of the nitroxide prevented measurement by *r_1_*.

**Table 4:** Characterization of HSA-NIT conjugates’ magnetic properties at 7 T.

HSA Type	t °C	*r_1_*, mM^−1^s^−1^	*r_2_*, mM^−1^s^−1^	n *	τ_cor_ (ns) **	*r_1_*, mM^−1^s^−1^	*r_2_*, mM^−1^s^−1^	r_2_/r_1_
		*per albumin molecule*				*per nitroxide radical*		
**HSA-NIT-8**	25	0.80 ±0.03	11.2 ±0.2	2.4	14/2.6	0.33	4.7	14.2
								
**HSA-NIT-7**	25	1.99 ±0.05	27.9 ±0.5	3.9	14/2.7	0.51	7.2	14.1
	37	1.51 ±0.03	20.6 ±0.1	3.9		0.39	5.3	13.6
								
**HSA-NIT-6**	25	1.86 ±0.04	20.4 ±0.3	3.9	10/2.1	0.48	5.2	10.8
	37	1.51 ±0.05	16.6 ±0.3	3.9		0.39	4.3	11.0
								
**HSA-NIT-5**	25	2.24 ±0.08	27.7 ±0.2	4.8	10/1.9	0.47	5.8	12.3

* Nitroxides per albumin molecule. ** «slow»/«fast» motional components at 25 °C.

## Supplementary Materials

The following are available online at Materials and Methods; Synthesis of Nitroxide Derivatives of Homocysteine Thiolactone; Synthesis and Characterization of HSA-Nitroxide Conjugates; Identification of *N*-Homocysteinylation Sites in the HSA-Nit Conjugates; The pK_A_ and Accessible Surface Area (ASA) of Lysine Residues; Reduction of HSA-Nit Conjugates; Relaxivity of HSA-Nit Conjugates; Cell Culture and Toxicity Assay; Infrared Spectra; NMR Spectra; and X-ray Data for **6A**, **13** and **16**.

## References

[1] Wahsner J., Gale E.M., Rodríguez-Rodríguez A., Caravan P. (2019). Chemistry of MRI contrast agents: Current challenges and new frontiers. Chem. Rev..

[2] Kim H.K., Lee G.H., Chang Y. (2018). Gadolinium as an MRI contrast agent. Future Med. Chem..

[3] Li H., Meade T.J. (2019). Molecular MR Imaging with Gd(III)-based Agents: Challenges and Key Advances. J. Am. Chem. Soc..

[4] Akakuru O.U., Iqbal M.Z., Saeed M., Liu C., Paunesku T., Woloschak G., Hosmane N.S., Wu A. (2019). The Transition from Metal-Based to Metal-Free Contrast Agents for T1 Magnetic Resonance Imaging Enhancement. Bioconjug. Chem..

[5] Thomsen H.S., Morcos S.K., Almén T., Bellin M.F., Bertolotto M., Bongartz G., Clement O., Leander P., Heinz-Peer G., Reimer P. (2013). Nephrogenic systemic fibrosis and gadolinium-based contrast media: Updated ESUR Contrast Medium Safety Committee guidelines. Eur. Radiol..

[6] Pasquini L., Napolitano A., Visconti E., Longo D., Romano A., Tomà P., Espagnet M.C.R. (2018). Gadolinium-Based Contrast Agent-Related Toxicities. CNS Drugs.

[7] Ramalho J., Ramalho M. (2017). Gadolinium Deposition and Chronic Toxicity. Magn. Reson. Imaging Clin. N. Am..

[8] Malikova H., Holesta M. (2017). Gadolinium contrast agents – are they really safe?. J. Vasc. Access.

[9] Swaminathan S., Horn T.D., Pellowski D., Abul-Ezz S., Bornhorst J.A., Viswamitra S., Shah S.V. (2007). Nephrogenic systemic fibrosis, gadolinium, and iron mobilization. N. Engl. J. Med..

[10] Abakumov M.A., Semkina A.S., Skorikov A.S., Vishnevskiy D.A., Ivanova A.V., Mironova E., Davydova G.A., Majouga A.G., Chekhonin V.P. (2018). Toxicity of iron oxide nanoparticles: Size and coating effects. J. Biochem. Mol. Toxicol..

[11] Singh N., Jenkins G.J.S., Asadi R., Doak S.H. (2010). Potential toxicity of superparamagnetic iron oxide nanoparticles (SPION). Nano Rev..

[12] Vallabani N.V.S., Singh S. (2018). Recent advances and future prospects of iron oxide nanoparticles in biomedicine and diagnostics. 3 Biotech.

[13] Tirotta I., Dichiarante V., Pigliacelli C., Cavallo G., Terraneo G., Bombelli F.B., Metrangolo P., Resnati G. (2015). 19F Magnetic Resonance Imaging (MRI): From Design of Materials to Clinical Applications. Chem. Rev..

[14] Jirak D., Galisova A., Kolouchova K., Babuka D., Hruby M. (2019). Fluorine polymer probes for magnetic resonance imaging: Quo vadis?. Magn. Reson. Mater. Physics Biol. Med..

[15] Nguyen H.V.T., Chen Q., Paletta J.T., Harvey P., Jiang Y., Zhang H., Boska M.D., Ottaviani M.F., Jasanoff A., Rajca A. (2017). Nitroxide-Based Macromolecular Contrast Agents with Unprecedented Transverse Relaxivity and Stability for Magnetic Resonance Imaging of Tumors. ACS Cent. Sci..

[16] Dharmarwardana M., Martins A.F., Chen Z., Palacios P.M., Nowak C.M., Welch R.P., Li S., Luzuriaga M.A., Bleris L., Pierce B.S. (2017). Nitroxyl Modified Tobacco Mosaic Virus as a Metal-Free High- Relaxivity MRI and EPR Active Superoxide Sensor. Physiol. Behav..

[17] Bye N., Hutt O.E., Hinton T.M., Acharya D.P., Waddington L.J., Moffat B.A., Wright D.K., Wang H.X., Mulet X., Muir B.W. (2014). Nitroxide-loaded hexosomes provide MRI contrast in vivo. Langmuir.

[18] M Davis R., B Mitchell J., C Krishna M. (2012). Nitroxides as Cancer Imaging Agents. Anti-Cancer Agents Med. Chem..

[19] Nguyen H.V.T., Detappe A., Gallagher N.M., Zhang H., Harvey P., Yan C., Mathieu C., Golder M.R., Jiang Y., Ottaviani M.F. (2018). Triply Loaded Nitroxide Brush-Arm Star Polymers Enable Metal-Free Millimetric Tumor Detection by Magnetic Resonance Imaging. ACS Nano.

[20] Soikkeli M., Horkka K., Moilanen J.O., Timonen M., Kavakka J., Heikkinen S. (2018). Synthesis, stability and relaxivity of teepo-met: An organic radical as a potential tumour targeting contrast agent for magnetic resonance imaging. Molecules.

[21] Soikkeli M., Sievänen K., Peltonen J., Kaasalainen T., Timonen M., Heinonen P., Rönkkö S., Lehto V.P., Kavakka J.S., Heikkinen S. (2015). Synthesis and in vitro phantom NMR and MRI studies of fully organic free radicals, TEEPO-glucose and TEMPO-glucose, potential contrast agents for MRI. RSC Adv..

[22] Chan H.C., Sun K.Q., Magin R.L., Swartz H.M. (1990). Potential of Albumin Labeled with Nitroxides as a Contrast Agent for Magnetic Resonance Imaging and Spectroscopy. Bioconjug. Chem..

[23] Lee H., Shahrivarkevishahi A., Lumata J.L., Luzuriaga M.A., Hagge L.M., Benjamin C.E., Brohlin O.R., Parish C.R., Firouzi H.R., Nielsen S.O. (2020). Chemical Science enhancement of metal-free magnetic resonance imaging contrast agents. Chem. Sci..

[24] Kocherginsky N., Swartz H.M. (1995). Metabolism and Distribution of Nitroxides In Vivo. Nitroxide Spin Labels: Reactions in Biology and Chemistry.

[25] Teucher M., Zhang H., Bader V., Winklhofer K.F., García-Sáez A.J., Rajca A., Bleicken S., Bordignon E. (2019). A new perspective on membrane-embedded Bax oligomers using DEER and bioresistant orthogonal spin labels. Sci. Rep..

[26] Paletta J.T., Pink M., Foley B., Rajca S., Rajca A. (2012). Synthesis and reduction kinetics of sterically shielded pyrrolidine nitroxides. Org. Lett..

[27] Kirilyuk I.A., Polienko Y.F., Krumkacheva O.A., Strizhakov R.K., Gatilov Y.V., Grigor’ev I.A., Bagryanskaya E.G. (2012). Synthesis of 2,5-bis(spirocyclohexane)-substituted nitroxides of pyrroline and pyrrolidine series, including thiol-specific spin label: An analogue of MTSSL with long relaxation time. J. Org. Chem..

[28] Soule B.P., Hyodo F., Matsumoto K.I., Simone N.L., Cook J.A., Krishna M.C., Mitchell J.B. (2007). The chemistry and biology of nitroxide compounds. Free Radic. Biol. Med..

[29] Rajca A., Wang Y., Boska M., Paletta J.T., Olankitwanit A., Swanson M.A., Mitchell D.G., Eaton S.S., Eaton G.R., Rajca S. (2012). Organic Radical Contrast Agents for Magnetic Resonance Imaging. J. Am. Chem. Soc..

[30] Chen C., Kang N., Xu T., Wang D., Ren L., Guo X. (2015). Core-shell hybrid upconversion nanoparticles carrying stable nitroxide radicals as potential multifunctional nanoprobes for upconversion luminescence and magnetic resonance dual-modality imaging. Nanoscale.

[31] Alvaradejo G.G., Nguyen H.V.-T., Harvey P., Gallagher N.M., Le D., Ottaviani M.F., Jasanoff A., Delaittre G., Johnson J.A. (2017). Polyoxazoline-Based Bottlebrush and Brush-Arm Star Polymers via ROMP: Syntheses and Applications as Organic Radical Contrast Agents. Physiol. Behav..

[32] Sleep D., Cameron J., Evans L.R. (2013). Albumin as a versatile platform for drug half-life extension. Biochim. Biophys. Acta.

[33] Caspersen M.B., Kuhlmann M., Saxton M.J., Cameron J. (2017). Albumin-based drug delivery using cysteine 34 chemical conjugates – important considerations and requirements. Ther. Deliv.

[34] Tormyshev A.V., Chubarov A., Krumkacheva O., Trukhin D., Rogozhnikova O., Spitsina A., Kuzhelev A., Koval V., Fedin M., Bowman M. (2020). A Methanethiosulfonate Derivative of OX063 Trityl: A Promising and Efficient Reagent for SDSL of Proteins. Chem. A Eur. J..

[35] Krumkacheva O.A., Timofeev I.O., Politanskaya L.V., Polienko Y.F., Tretyakov E.V., Rogozhnikova O.Y., Trukhin D.V., Tormyshev V.M., Chubarov A.S., Bagryanskaya E.G. (2019). Triplet Fullerenes as Prospective Spin Labels for Nanoscale Distance Measurements by Pulsed Dipolar EPR. Angew. Chemie Int. Ed..

[36] Spicer C.D., Davis B.G. (2014). Selective chemical protein modification. Nat. Commun..

[37] Matos M.J., Oliveira B.L., Martínez-Sáez N., Guerreiro A., Cal P.M.S.D., Bertoldo J., Maneiro M.M., Perkins E., Howard J., Deery M.J. (2018). Chemo- and Regioselective Lysine Modification on Native Proteins. J. Am. Chem. Soc..

[38] Asano S., Patterson J.T., Gaj T., Barbas C.F. (2014). Site-selective labeling of a lysine residue in human serum albumin. Angew. Chemie Int. Ed..

[39] Chubarov A.S., Shakirov M.M., Koptyug I.V., Sagdeev R.Z., Knorre D.G., Godovikova T.S. (2011). Synthesis and characterization of fluorinated homocysteine derivatives as potential molecular probes for 19F magnetic resonance spectroscopy and imaging. Bioorg. Med. Chem. Lett..

[40] Lisitskiy V.A., Khan H., Popova T.V., Chubarov A.S., Zakharova O.D., Akulov A.E., Shevelev O.B., Zavjalov E.L., Koptyug I.V., Moshkin M.P. (2017). Multifunctional human serum albumin-therapeutic nucleotide conjugate with redox and pH-sensitive drug release mechanism for cancer theranostics. Bioorganic Med. Chem. Lett..

[41] Sikora M., Marczak Ł., Kubalska J., Graban A., Jakubowski H. (2014). Identification of N-homocysteinylation sites in plasma proteins. Amino Acids.

[42] Marczak L., Sikora M., Stobiecki M., Jakubowski H. (2011). Analysis of site-specific N-homocysteinylation of human serum albumin in vitro and in vivo using MALDI-ToF and LC-MS/MS mass spectrometry. J. Proteomics.

[43] Sikora M., Marczak Ł., Twardowski T., Stobiecki M., Jakubowski H. (2010). Direct monitoring of albumin lysine-525 N-homocysteinylation in human serum by liquid chromatography/mass spectrometry. Anal. Biochem..

[44] Präbst K., Engelhardt H., Ringgeler S., Hübner H. (2017). Basic Colorimetric Proliferation Assays: MTT, WST, and Resazurin. Cell Viability Assays. Methods in Molecular Biology.

[45] Mosmann T. (1983). Rapid colorimetric assay for cellular growth and survival: Application to proliferation and cytotoxicity assays. J. Immunol. Methods.

[46] Jakubowski H. (2004). Molecular basis of homocysteine toxicity in humans. Cell. Mol. Life Sci..

[47] Chubarov A.S., Zakharova O.D., Koval O.A., Romaschenko A.V., Akulov A.E., Zavjalov E.L., Razumov I.A., Koptyug I.V., Knorre D.G., Godovikova T.S. (2015). Design of protein homocystamides with enhanced tumor uptake properties for 19F magnetic resonance imaging. Bioorg. Med. Chem..

[48] Sibrian-Vazquez M., Escobedo J.O., Lim S., Samoei G.K., Strongin R.M. (2010). Homocystamides promote free-radical and oxidative damage to proteins. Proc. Natl. Acad. Sci. USA.

[49] Frank D., Espeel P., Claessens S., Mes E., Du Prez F.E. (2016). Synthesis of thiolactone building blocks as potential precursors for sustainable functional materials. Tetrahedron.

[50] Espeel P., Prez F. (2015). One-pot double modification of polymers based on thiolactone chemistry. Adv. Polym. Sci..

[51] Dobrynin S.A., Khoroshunova Y.V., Kirilyuk I.A. (2019). Synthesis of 2,2,5,5-tetraethyl-3-carboxypyrrolidine 1-oxyl. RU. Patent No..

[52] Dichtl A., Seyfried M., Schoening K.U. (2008). A novel method for the synthesis of N-alkoxyamines starting from nitroxide radicals and ketones. Synlett.

[53] Sheldrick G.M. (2015). Crystal structure refinement with SHELXL. Acta Cryst..

[54] Dobrynin S.A., Glazachev Y.I., Gatilov Y.V., Chernyak E.I., Salnikov G.E., Kirilyuk I.A. (2018). Synthesis of 3,4-Bis(hydroxymethyl)-2,2,5,5-tetraethylpyrrolidin-1-oxyl via 1,3-Dipolar Cycloaddition of Azomethine Ylide to Activated Alkene. J. Org. Chem..

[55] Kálai T., Bognár B., Zsolnai D., Berente Z., Hideg K. (2012). Synthesis of nitroxide-annulated carbocycles and heterocycles. Synthesis.

[56] Kalai T., Balog M., Jekő J., Hideg K. (1999). Synthesis and reactions of a symmetric paramagnetic pyrrolidine diene. Synthesis.

[57] Janatova J., Fuller J.K., Hunter M.J. (1968). The heterogeneity of bovine albumin with respect to sulfhydryl and dimer content. J. Biol. Chem..

[58] Sterling H.J., Prell J.S., Cassou C.A., Williams E.R. (2011). Protein conformation and supercharging with DMSO from aqueous solution. J. Am. Soc. Mass Spectrom..

[59] De Abreu Costa L., Ottoni M.H.F., Dos Santos M.G., Meireles A.B., De Almeida V.G., De Fátima Pereira W., De Avelar-Freitas B.A., Brito-Melo G.E.A. (2017). Dimethyl sulfoxide (DMSO) decreases cell proliferation and TNF-α, IFN-, and IL-2 cytokines production in cultures of peripheral blood lymphocytes. Molecules.

[60] Yiannios C.N., Karabinos J.V. (1963). Oxidation of Thiols by Dimethyl Sulfoxide. J. Org. Chem..

[61] Papanyan Z.K. (2013). Interaction of L-cysteine with dimethyl sulfoxide in mild conditions. Proc. Yerevan State Univ..

[62] Perła-Kaján J., Twardowski T., Jakubowski H., Perla-Kajan J., Twardowski T., Jakubowski H. (2007). Mechanisms of homocysteine toxicity in humans. Amino Acids.

[63] Jakubowski H. (2013). Homocysteine in Protein Structure/Function and Human Disease.

[64] Perczel A., Hollósi M., Tusnády G., Fasman G.D. (1991). Convex constraint analysis: A natural deconvolution of circular dichroism curves of proteins. Protein Eng..

[65] Varkovitzky R.L. (2012). Assimilation, accommodation, and overaccommodation: An examination of information processing styles in female victims of adolescent and adult sexual assault. ProQuest Diss. Theses.

[66] Paoli P., Sbrana F., Tiribilli B., Caselli A., Pantera B., Cirri P., De Donatis A., Formigli L., Nosi D., Manao G. (2010). Protein N-homocysteinylation induces the formation of toxic amyloid-like protofibrils. J. Mol. Biol..

[67] Willard L., Ranjan A., Zhang H., Monzavi H., Boyko R.F., Sykes B.D., Wishart D.S. (2003). VADAR: A web server for quantitative evaluation of protein structure quality. Nucleic Acids Res..

[68] Olsson M.H.M., Søndergaard C.R., Rostkowski M., Jensen J.H. (2011). PROPKA3: Consistent Treatment of Internal and Surface Residues in Empirical pKa Predictions. J. Chem. Theory Comput..

[69] Søndergaard C.R., Olsson M.H.M., Rostkowski M., Jensen J.H. (2011). Improved treatment of ligands and coupling effects in empirical calculation and rationalization of pKa values. J. Chem. Theory Comput..

[70] Altenbach C., Marti T., Khorana H.G., Hubbell W.L. (1990). Transmembrane protein structure: Spin labeling of bacteriorhodopsin mutants. Science.

[71] Stoll S., Schweiger A. (2007). Easyspin: Simulating CW ESR spectra. Biol. Magn. Reson..

[72] Stoll S., Schweiger A. (2006). EasySpin, a comprehensive software package for spectral simulation and analysis in EPR. J. Magn. Reson..

[73] Zhurko I.F., Dobrynin S., Gorodetskii A.A., Glazachev Y.I., Rybalova T.V., Chernyak E.I., Asanbaeva N., Bagryanskaya E.G., Kirilyuk I.A. (2020). 2-Butyl-2-tert-butyl-5,5-diethylpyrrolidine-1-oxyls: Synthesis and properties. Molecules.

[74] Jagtap A.P., Krstic I., Kunjir N.C., Hänsel R., Prisner T.F., Sigurdsson S.T. (2015). Sterically shielded spin labels for in-cell EPR spectroscopy: Analysis of stability in reducing environment. Free Radic. Res..

[75] Goldstein S., Merenyi G., Russo A., Samuni A. (2003). The role of oxoammonium cation in the SOD-mimic activity of cyclic nitroxides. J. Am. Chem. Soc..

[76] Sowers M.A., Mccombs J.R., Wang Y., Paletta J.T., Morton S.W., Dreaden E.C., Boska M.D., Ottaviani M.F., Hammond P.T., Rajka A. (2014). Redox-responsive branched-bottlebrush polymers for in vivo MRI and fluorescence imaging Molly. Nat Commun..

[77] Popova T.V., Khan H., Chubarov A.S., Lisitskiy V.A., Antonova N.M., Akulov A.E., Shevelev O.B., Zavjalov E.L., Silnikov V.N., Ahmad S. (2018). Biotin-decorated anti-cancer nucleotide theranostic conjugate of human serum albumin: Where the seed meets the soil?. Bioorganic Med. Chem. Lett..

[78] Larsen M.T., Kuhlmann M., Hvam M.L., Howard K.A. (2016). Albumin-based drug delivery: Harnessing nature to cure disease. Mol. Cell. Ther..

[79] Levitt D.G., Levitt M.D. (2016). Human serum albumin homeostasis: A new look at the roles of synthesis, catabolism, renal and gastrointestinal excretion, and the clinical value of serum albumin measurements. Int. J. Gen. Med..

