# Supplementary material for: Human Serum Albumin Labelled with Sterically-Hindered Nitroxides as Potential MRI Contrast Agents
Source: Molecules. 2020 Apr 8;25(7):1709. doi: 10.3390/molecules25071709 (PMC7180620; doi:10.3390/molecules25071709)
Supplement: Supplementary file 1 [file molecules-25-01709-s001.pdf]

Human serum albumin labelled with sterically-hindered nitroxides as potential MRI contrast agents

Sergey Dobrynin[1], Sergei Kutseikin[2], Denis Morozov[1], Olesya Krumkacheva[3], Anna Spitsyna[1], Yurii Gatilov[1], Vladimir Silnikov[2], Goran Angelovski[4], Michael K. Bowman[1,5,*], Igor Kirilyuk[1,*] and Alexey Chubarov[2,*]

[1] N.N. Vorozhtsov Institute of Organic Chemistry SB RAS 630090 Novosibirsk, Russia;
[2] Institute of Chemical Biology and Fundamental Medicine SB RAS 630090 Novosibirsk, Russia;
[3] International Tomography Center SB RAS 630090 Novosibirsk, Russia;
[4] Max Planck Institute for Biological Cybernetics 72076 Tubingen, Germany;
[5] University of Alabama, Tuscaloosa, Alabama 35487-0336, United States;

* Correspondence: mkbowman@ua.edu (M.K.B.); chubarovalesha@mail.ru, Tel.: +7-913-763-1420 (A.C.); kirilyuk@nioch.nsc.ru Tel.: +7-383-330-7387 (I.K.)

1. MATERIALS AND METHODS 1
2. SYNTHESIS OF NITROXIDE DERIVATIVES OF HOMOCYSTEINE THIOLACTONE 4
2.1. 3,4-Bis-(methanesulfonyloxymethyl)-2,2,5,5-tetraethylpyrrolidin-1-oxyl (10) 4
2.2. 3,4-Bis-(bromomethyl)-2,2,5,5-tetraethylpyrrolidin-1-oxyl (11) 4
2.3. 2,2,5,5-Tetraethyl-3,4-dimethylidenepyrrolidin-1-oxyl (12) 4
2.4. 1-Hydroxy-3,4-dimethylidene-2,2,5,5-tetraethylpyrrolidin hydrochloride (13) 4
2.5. 3,4-Bis(bromomethyl)-2,2,5,5-tetraethyl-2,5-dihydro-1*H*-pyrrol-1-oxyl (14) 5
2.6. 3,4-Bis-[(acetyloxy)methyl]-2,2,5,5-tetraethyl-2,5-dihydro-1*H*-pyrrol-1-oxyl (15) 5
2.7. 2,2,5,5-Tetraethyl-3,4-bis(hydroxymethyl)-2,5-dihydro-1*H*-pyrrol-1-oxyl (16) 5
2.8. 3,4-Dicarboxy-2,2,5,5-tetraethyl-2,5-dihydro-1*H*-pyrrol-1-oxyl (4) 6
2.9. 3-Carboxy-2,2,5,5-tetraethyl-4-[(2-oxothiolan-3-yl)carbamoyl]-2,5-dihydro-1*H*-pyrrol-1-oxyl (8) 6
2.10. 2,2,5,5-tetramethyl-3-[(2-oxothiolan-3-yl)carbamoyl]-2,5-dihydro-1*H*-pyrrol-1-oxyl (5) 7
2.11. 2,2,5,5-Tetramethyl-3-[(2-oxothiolan-3-yl)carbamoyl]pyrrolidin-1-oxyl (6) 7
2.12. 2,2,5,5-Tetraethyl-3-[(2-oxothiolan-3-yl)carbamoyl]pyrrolidin-1-oxyl (7) 8
3. SYNTHESIS AND CHARACTERIZATION OF HSA-NITROXIDE CONJUGATES 8
Synthesis of human serum albumin modified by nitroxide radical homocysteine thiolactone derivatives 8
Table S1. Dependence of modification degree calculated by measuring SH-group content in albumin conjugate and by MALDI TOF data 9
Table S2. Modification degree calculated by MALDI TOF and EPR data for HSA-NIT conjugates 9
Table S3. The parameters for EPR simulations in Fig. 3 10
4. IDENTIFICATION OF *N*-HOMOCYSTEINYLATION SITES IN THE HSA-NIT CONJUGATES 10
Table S4. Identification of specific *N*-homocysteinylation sites in HSA-NIT conjugates 11
5. THE P*K*A AND ACCESSIBLE SURFACE AREA (ASA) OF LYSINE RESIDUES 14
Table S5. Computer modelling (pK_a, ASA) of usually *N*-homocysteinylated Lys residues in HSA 14
Table S6. Computer modelling (pK_a, ASA) of all Lys residues in HSA 14
6. REDUCTION OF HSA-NIT CONJUGATES 15
Table S7. T_1-relaxation time measurements under reducing conditions for HSA-NIT conjugates 16
7. RELAXIVITY (R_1 AND R_2) OF HSA-NIT CONJUGATES 17
8. CELL CULTURE AND TOXICITY ASSAY 18
9. INFRARED SPECTRA 18
10. NMR SPECTRA 21
11. X-RAY DATA FOR 6A, 13 AND 16 COMPOUNDS 22
REFERENCES 23

1. Materials and methods

Materials. The human serum albumin (HSA) used in this study was purchased from Sigma–Aldrich Chem. Co. (A3782, USA). Ellman's test indicated that the albumin preparation contained 0.29±0.05 sulfhydryl groups per protein molecule. MS

(MALDI ToF) m/z HSA 66.48 kDa. 5,5'-dithio-bis(2-nitrobenzoic acid) (DTNB), DL-homocysteine thiolactone (HTL) hydrochloride and all solvents and other reagents, unless stated differently, were purchased from Sigma at the highest available grade and used without purification. Cascade Blue ethylenediamine trisodium salt and MTT (3-[4,5-dimethylthiazol-2-yl]-2,5-diphenyltetrazolium bromide) assay kit were purchased from Invitrogen. Centricon concentrators with 3 kDa molecular weight cut-off were purchased from Millipore.

Nitroxides **1**, **2** and **9** were prepared according to literature protocols [1,2]. 3-Carboxy-2,2,5,5-tetraethylpyrrolidin-1-oxyl (**3**) was prepared according to [1], its spectral parameters and analytical data correspond to the literature data [5]. 3-Carboxy-2,2,5,5-tetraethylpyrrolidin-1-oxyl (**1**) was prepared as described in patent [1], IR spectrum of the nitroxide is given in Fig. S9.1., the ^{1}H NMR spectrum of the corresponding hydroxylamine trifluoroacetate is given in Fig. S10.1.

EPR. Samples for EPR were placed in glass capillary tubes (OD 1.5 mm, ID 0.9 mm). Continuous wave (CW) EPR measurements at X-band were performed at room temperature on an X-band Bruker EMX spectrometer. Experimental CW EPR settings were as follows: central field 351.48 mT, sweep width 15 mT, microwave power 6.377 mW, microwave frequency 9.87 GHz, modulation frequency 100 kHz, modulation amplitude 0.15 mT, conversion time 81.92 ms, number of points 1024, number of scans 10. Double integrals were calculated with Bruker WinEPR software. Spin concentration was determined by comparing spectral double integrals of the sample and a solution with known concentration. The experimental spectra were simulated using EasySpin software [6,7]. All spectra were simulated in the slow-motion regime with the EasySpin chili function.

Experimental EPR spectra were decomposed using EasySpin software into three components with different rotational correlation times – “slow”, “fast” and “free”. The rotational correlation time shows mobility of the label: longer times correspond to more immobilized labels and *vice versa*. The g-tensor g_{ii} and hyperfine tensor A_{xx}, A_{yy} values were the same in all spectra: g_{xx}=2.009, g_{yy}=2.006, g_{zz}=2.0025, A_{xx}=A_{yy}=0.5; the values of A_{zz} were varied. Line broadenings were calculated assuming Voigtian line shape – convolution of Lorentzian and Gaussian line shape.

Mass spectra of proteins were recorded on Bruker Autoflex Speed (Bruker, Germany) MALDI-TOF mass spectrometer in a positive linear mode. A smartbeam-II laser was used with 2,5-Dihydroxyacetophenone (2,5-DHAP) as the matrix. Protein samples were desalted by ZipTip C4 pipette tips. A 2 µL protein sample solution was mixed with 2 µL of a 2 % TFA (trifluoroacetic acid). To the latter solution 2 µL of the matrix (2,5-DHAP) was added. The mixture was pipetted up and down until the crystallization started. Mass spectra were obtained by averaging 3000 laser shots. External calibration was provided by $[M+H]^+$ peak of human serum albumin at *m/z* 66467. MALDI ToF data was deconvoluted using the software package mMass (http://www.mmass.org).

HRMS were recorded on double-focusing, high-resolution mass spectrometer equipped with a high-performance toroidal ESA and quadrupole time-of-flight mass spectrometer.

Circular dichroism (CD) data were collected at 25 °C with a JASCO J-600 spectrophotometer with time constant 4 s, bandwidth 1 nm, using a 0.01 cm path length quartz cell. All CD spectra were performed in triplicate from 185 to 240 nm, and the averaged values are presented. In order to determine the percentages of α-helices, β-sheets, turns, and the disordered structures, we minimized the difference between the theoretical and experimental curves. The theoretical curves were calculated as a linear combination of the basis spectra of various components of the secondary structures taken from the CCA+ software [8].

SDS-PAGE. Human serum albumin conjugates were analyzed by sodium dodecyl sulfate polyacrylamide gel electrophoresis using 7 % PAAG under Laemmli condition without the addition of dithiothreitol (DTT) with subsequent Coomassie Brilliant Blue staining. Quantitative data were obtained by digitizing the gel using GelPro Analyzer software.

NMR spectra were recorded at 25 °C in 5 mm NMR sample tubes. The chemical shifts were expressed in parts per million, ppm (δ). All ^{1}H chemical shifts were calculated relative to the residual ^{1}H NMR signal of the deuterated NMR solvent. The spin-spin coupling constants (J) are reported in hertz (Hz) and spin multiples are given as s (singlet), d (doublet), t (triplet), q (quartet), m (multiplet).

Electronic absorption spectra were recorded on a UV-1800 spectrometer (Shimadzu, Japan). The concentrations of HSA solutions were determined by absorption at 278 nm, pH 7.4 (PBS), using the molar extinction coefficient $\varepsilon = 3.7\times10^4$ M^{-1}cm^{-1}.

IR spectra were acquired on a Bruker Tensor 27 FTIR spectrometer in KBr or neat and are reported in wavenumbers (cm^{-1}).

TLC was performed on DC-Alufolien Kieselgel 60 F_{254} (Merck, Darmstand, Germany). Reactions were monitored by TLC and carried out using UV light at 254 nm, 1 % aqueous permanganate, 10 % solution of phosphomolybdic acid in ethanol, and/or Dragendorff reagent as visualizing agents. Column chromatography was performed on silica gel 60 (70−230 mesh).

The number of thiol groups per albumin molecule was determined using Ellman's method as described in the literature [9] or on the Thermo Scientific website and employed 5,5'-Dithio-bis(2-nitrobenzoic acid) (DTNB) at pH 8 at 412 nm (ε = 1.4 × 10^4 M^{-1} cm^{-1}) with a UV-1800 spectrometer using unmodified HSA as control. DTNB produces a measurable yellow-colored product when it reacts with free SH groups.

MRI measurements were performed on Bruker 7 T BioSpec 70/30 USR magnet (software version Paravision 5.1) using Bruker volume coil (RF RES 300 1H 075/040 QSN TR). T_1-weighted images where acquired using the Fast Low Angle Single Shot (FLASH) pulse sequence, and T_2-weighted images using the Multi-Slice Multi-Echo (MSME) pulse sequence. Phantoms consisted of 9 vials containing PBS and four HSA-nitroxide conjugates (0.2 mM and 0.5 mM); Acquisition parameters: T_1-weighted: Field of view (FOV)= 31.8 x 31.8 mm2, matrix size (MTX)= 212 x 212, slice thickness 0.5 mm, flip angle (FA)= 90 0, repetition time (TR)=615 ms, echo time (TE)= 2.746, number of excitations (NEX)= 10, and total acquisition time (TA)= 21 min 44 sec; T_2-weighted: FOV=32.0 x 32.0 mm2, MTX 160 x 160, slice thickness 0.5 mm, TR=10000 ms, TE=80 ms, NEX= 4, TA=1 h 46 min 40 sec.

2. Synthesis of nitroxide derivatives of homocysteine thiolactone

2.1. 3,4-Bis-(methanesulfonyloxymethyl)-2,2,5,5-tetraethylpyrrolidin-1-oxyl (10)

Methanesulfonyl chloride (1.41 g, 10.8 mmol) was added in one portion to a stirred solution of 2,2,5,5-tetraethyl-3,4-bis(hydroxymethyl)pyrrolidin-1-oxyl (**9**) (1.27 g, 4.9 mmol) in dry chloroform (35 mL). Then DIPEA (1.39 g, 10.8 mmol) was added in one portion to the stirred solution. The reaction mixture was placed into a cold-water bath for 30 min and then stirred at room temperature. When the reaction was complete (controlled with TLC, Silicagel, hexane – ethyl acetate mixture = 1:1), the mixture was washed with brine and then the organic phase was dried with Na_2SO_4. The solvent was distilled off under reduced pressure. The residue was used in the next step without any purification.

2.2. 3,4-Bis-(bromomethyl)-2,2,5,5-tetraethylpyrrolidin-1-oxyl (11)

3,4-Bis-(methanesulfonyloxymethyl)-2,2,5,5-tetraethylpyrrolidin-1-oxyl **10** (16.37 g, 39.5 mmol) was dissolved in dry THF (250 mL) and solid LiBr (13.72 g, 158 mmol) was added in one portion. The mixture was stirred under reflux for 10 h. The end of reaction was controlled with TLC (Silicagel, hexane – ethyl acetate mixture = 1:1). After cooling the reaction mixture to room temperature, the THF was diluted with Et_2O (200 ml) and washed with brine (3×150 ml). The organic phase was dried with Na_2SO_4. The solvent was distilled off under reduced pressure and the residue was recrystallized from hexane to give **11** (11.2 g, 74 % from **9**): yellow crystals, mp 131–132 °C; UV (EtOH) $\lambda_{max/nm}$ (*lg*ε, $M^{-1}cm^{-1}$): 212 (3.28), 240 (3.24). IR ν_{max} 3037, 2972, 2933, 2877, 1462, 1437, 1415, 1383, 1346, 1275, 1157, 929, 833, 731, 671, 627, 563, 497, 432. Anal. Calcd for $C_{14}H_{26}NOBr_2$: C, 43.77; H, 6.82; N, 3.65; Br, 41.60. Found: C, 43.90; H, 6.52; N, 3.64; Br, 41.73. HRMS EI (m/z) calcd for $C_{14}H_{26}ON^{79}Br_2$ 382.0376, found 382.038.

2.3. 2,2,5,5-Tetraethyl-3,4-dimethylidenepyrrolidin-1-oxyl (12)

A solution of NaOH (8.76 g, 219 mmol) in water (80 mL) was added to a solution of **11** (15.3 g, 39.8 mmol) in ethanol (150 mL), and the mixture was stirred under reflux for 3 h. The end of the reaction was controlled with TLC (Silicagel, hexane – ethyl acetate mixture = 9:1). After cooling to room temperature, the mixture was extracted with hexane. The organic extract was dried with Na_2SO_4 and evaporated under reduced pressure. The residue was separated using column chromatography on silicagel, hexane – ethyl acetate mixture 9:1 to give **12** (7.5 g, 85 %): yellow oil; UV (EtOH) $\lambda_{max/nm}$ (*lg*ε, $M^{-1}cm^{-1}$): 237 (3.84). IR ν_{max} 2968, 2939, 2879, 1462, 1410, 1379, 1009, 953, 924, 877, 858, 818. Anal. Calcd for $C_{14}H_{24}NO$: C, 75.63; H 10.88; N, 6.30. Found: C, 74.80; H, 10.82; N, 6.39. HRMS EI (m/z) calcd for $C_{14}H_{24}NO$ 222.1852, found 222.1855.

2.4. 1-Hydroxy-3,4-dimethylidene-2,2,5,5-tetraethylpyrrolidin hydrochloride (13)

Dry hydrogen chloride was bubbled through a solution of **12** (6.6 g, 29.7 mmol) in isopropanol (150 mL) until the solution color became slightly yellow. The solution stayed for 24 h at room temperature. Then, the solvent was distilled off under reduced pressure, and the residue was washed with ether and dried at room temperature. Yield: 6.8 g (88 %), colorless crystals,

mp 138.5–139.7 °C; UV (EtOH) $\lambda_{max/nm}$ (*lg*ε, $M^{-1}cm^{-1}$): 247 (3.78). ^1H NMR (500 MHz; $CDCl_3$, δ): 0.88 (t, Jt=7.4 Hz, 6H), 1.01 (t, Jt=7.4 Hz, 6H), 1.62 (qd, Jq=7.4 Hz, Jd= 14.6 Hz, 2H), 1.87-2.08(m, 6H), 4.85 (s, 2H), 5.65 (s, 2H), 9.8 (br, 1H), 10.56 (d, Jd=6.5 Hz, 1H). ^{13}C NMR (125 MHz; $CDCl_3$, δ): 7.7, 7.9, 26.3, 27.1, 80.2, 109.9, 142.9. IR ν_{max} 3431, 3088, 2970, 2943, 2885, 2804, 2727, 2686, 2632, 2578, 2517, 2490, 1616, 1493, 1454, 1406, 1394, 922, 752, 669. Anal. Calcd for $C_{14}H_{26}NOCl$: C, 64.72; H 10.09; N, 5.39; Cl, 13.64. Found: C, 65.04; H, 9.88; N, 5.45; Cl, 13.53. Crystal data (Fig. S11.1): $C_{14}H_{26}NOCl$, M = 259.81, triclinic, space group *P*-1, at 296 K: a = 9.1498(13), b = 9.3495(14), c = 10.0713(14) Å, α = 72.725(7), β = 86.532(7), γ = 64.995(7)°, V = 743.48(19) Å^3, Z = 2, d_{calc} = 1.161 g/cm^3, μ = 0.244 mm^{-1}, a total of 23455 I (θ_{max} = 29.16°), 4000 unique (R_{int} = 0.0283), 3580 I > 2σ(I), 166 parameters. GooF = 0.991, R_1 = 0.0378, wR_2 = 0.1008 [I > 2σ(I)], R_1 = 0.0426, wR_2 = 0.1066 (all data), max/min diff. peak 0.361/-0.193 e·Å^{-3}.

2.5. 3,4-Bis(bromomethyl)-2,2,5,5-tetraethyl-2,5-dihydro-1*H*-pyrrol-1-oxyl (14)

A solution of Br_2 (9.36 g, 58.6 mmol) in dry chloroform (80 mL) was added dropwise to a stirred solution of **13** (7.25 g, 27.9 mmol) in dry chloroform (100 mL). After bromine was added, the mixture was stirred under reflux for 3 h. The end of the reaction was controlled with TLC (Silicagel, hexane – ethyl acetate mixture = 1:1). After cooling the reaction mixture to room temperature, a solution of $NaNO_2$ (5.78 g, 84 mmol) in water (50 mL) was added and the mixture was stirred at room temperature for 20 min. The mixture was washed with a solution of $Na_2S_2O_3$ (20 %) in water, and then the organic phase was dried with Na_2SO_4. The solvent was distilled off under reduced pressure to give **14** (7.99 g, 75 %): dark yellow crystals, mp 43.4–43.8 °C; UV (EtOH) $\lambda_{max/nm}$ (*lg*ε, $M^{-1}cm^{-1}$): 234 (4.0). IR ν_{max} 3030, 2981, 2966, 2927, 2875, 2848, 1641, 1460, 1444, 1375, 1336, 1225, 1200, 926, 899, 827, 737, 638, 586, 559. Anal. Calcd for $C_{14}H_{24}NOBr_2$: C, 44.00; H 6.33; N, 3.67; Br, 41.82. Found: C, 44.90; H, 6.40; N, 3.71; Br, 42.13. HRMS EI (m/z) calcd for $C_{14}H_{24}NO^{79}Br_2$ 380.0219, found 380.0214.

2.6. 3,4-Bis-[(acetyloxy)methyl]-2,2,5,5-tetraethyl-2,5-dihydro-1*H*-pyrrol-1-oxyl (15)

A solution of **14** (782 mg, 2.05 mmol) and NaOAc (729 mg, 8.9 mmol) in DMF (10 mL) and water (4 mL) was stirred under reflux for 4 h. The end of the reaction was controlled with TLC (Silicagel, hexane – ethyl acetate mixture = 4:1). After cooling the reaction mixture to room temperature, the mixture was salted and extracted with hexane five times. The organic phase was dried with Na_2SO_4, and then the solvent was distilled off under reduced pressure. The residue was separated using column chromatography on silicagel, hexane – ethyl acetate mixture 4:1 to give **15** (488 mg, 70 %): yellow oil; IR ν_{max} 2970, 2939, 2881, 1745, 1458, 1381, 1365, 1232, 1026, 966, 604, 553. Anal. Calcd for $C_{18}H_{30}NO_5$: C, 63.51; H, 8.88; N, 4.11. Found: C, 63.52; H 8.64; N, 4.18. HRMS EI (m/z) calcd for $C_{18}H_{30}NO_5$ 340.2118, found 340.2121.

2.7. 2,2,5,5-Tetraethyl-3,4-bis(hydroxymethyl)-2,5-dihydro-1*H*-pyrrol-1-oxyl (16)

A concentrated solution of ammonia in water (5 mL) was added to a solution of **15** (488 mg, 1.43 mmol) in methanol (5 mL) and then the mixture was stirred for 24 h. The end of the reaction was controlled with TLC (Silicagel, hexane – ethyl acetate

mixture = 1:2). After the end of the reaction, the solvent was distilled off under reduced pressure and the residue was separated using column chromatography on silicagel, hexane – ethyl acetate mixture 1:2 to give **16** (329 mg, 91 %): yellow crystals, mp 96.9–97.6 °C; UV (EtOH) $\lambda_{max/нм}$ (*lg*ε, $M^{-1}cm^{-1}$): 231 (3.45). IR ν_{max} 3460, 2968, 2939, 2879, 1658, 1461, 1404, 1211, 1090, 1041, 1016, 995, 937, 864, 825, 644, 569, 546. Anal. Calcd for $C_{14}H_{26}NO_3$: C, 65.59; H, 10.22; N, 5.46. Found: C, 65.49; H 10.23; N, 5.50. HRMS EI (m/z) calcd for $C_{14}H_{26}NO_3$ 256.1907, found 256.1908. Crystal data (Fig. S11.1): $C_{14}H_{26}NO_3$, M = 256.36, monoclinic, space group $P2_1/n$, at 296 K: a = 8.3141(7), b = 14.8748(15), c = 12.2606(12) Å, β = 109.427(4)°, V = 1429.9(2) Å^3, Z = 4, d_{calc} = 1.191 g/cm^3, μ = 0.082 mm^{-1}, a total of 27688 I (θ_{max} = 26.15°), 2840 unique (R_{int} = 0.0536), 2093 I > 2σ(I), 174 parameters. GooF = 1.052, R_1 = 0.0535, wR_2 = 0.1443 [I > 2σ(I)], R_1 = 0.0761, wR_2 = 0.1615 (all data), max/min diff. peak 0.661/-0.284 e·Å^{-3}.

2.8. 3,4-Dicarboxy-2,2,5,5-tetraethyl-2,5-dihydro-1*H*-pyrrol-1-oxyl (4)

To the solution of **16** (329 mg, 1.29 mmol) in benzene (17 mL) activated MnO_2 (6.5 g, 75 mmol) was added in one portion, and then the suspension was stirred for 2 h at room temperature. The end of the reaction was controlled with TLC (Silicagel, hexane – ethyl acetate mixture = 9:1). The MnO_2 was filtered off. The solvent was distilled off under reduced pressure, the residue was dissolved in *tert*-butanol (10 mL) and 2-methylbut-2-ene (4 g, 57 mmol), and then a solution of $NaClO_2$ (1.48 g, 16.4 mmol) and KH_2PO_4 (2.14 g, 15.7 mmol) in water (70 mL) was added. The mixture was stirred for 5 h, and then was alkalized by NaOH to pH 10 and extracted with hexane. The water phase was separated and acidified to pH 3 by $NaHSO_4$, and the solution was extracted with ether. The organic phase was dried with Na_2SO_4 and the solvent was distilled off under reduced pressure to give **4** (147 mg, 40 %): yellow crystals, mp 173.4 °C, the subsequent decomposition; UV (EtOH) $\lambda_{max/nm}$ (*lgε*, $M^{-1}cm^{-1}$): 224 (4.0). IR ν_{max} 3400, 2972, 2943, 2883, 1740, 1693, 1633, 1460, 1427, 1398, 1388, 1294, 1254, 1205, 1174, 1093, 1066, 1020, 933, 874, 804, 744, 6852. Anal. Calcd for $C_{14}H_{22}NO_5$: C, 59.14; H, 7.80; N, 4.93. Found: C, 58.72; H 7.52; N, 4.88. HRMS EI (m/z) calcd for $C_{14}H_{22}NO_5$. 284.1492, found 284.1495.

2.9. 3-Carboxy-2,2,5,5-tetraethyl-4-[(2-oxothiolan-3-yl)carbamoyl]-2,5-dihydro-1*H*-pyrrol-1-oxyl (8)

Sodium acetate (176 mg, 2.15 mmol) was added to a solution of **4** (147 mg, 0.52 mmol) in a mixture of dry chloroform (3 mL) and acetic anhydride (110 mg, 1.1 mmol) and refluxed for 4 h. The end of the reaction was controlled with TLC (Silicagel, methanol – ethyl acetate– acetic acid = 1:9:0.01). After cooling of the reaction mixture to room temperature, the mixture was washed with brine, and the organic phase was dried with Na_2SO_4 and the solvent was distilled off under reduced pressure to give the cyclic anhydride **17**. The residue was dissolved in dry chloroform (3 mL) and homocysteine thiolactone hydrochloride (95 mg, 0.62 mmol) was added in one portion. Then the solution of triethylamine (353 mg, 3.5 mmol) in dry chloroform (3 mL) was added dropwise to a cold stirred reaction mixture. The mixture was stirred for 30 min at room temperature. The end of reaction was controlled with TLC (Silicagel, hexane – ethyl acetate mixture = 9:1). After stirring the mixture was extracted with alkalized water (pH 10). The water phase was acidified to pH 3 and extracted with chloroform. The organic phase was dried

with Na_2SO_4 and the solvent was distilled off under reduced pressure. The residue was separated using column chromatography (silicagel, methanol:ethyl acetate:acetic acid = 1:9:0.01) to give **8** (150 mg, 75 %): yellow crystals, mp 107.8–108.9 °C; IR ν_{max} 3284, 3070, 2972, 2937, 2881, 1712, 1655, 1624, 1541, 1458, 1417, 1383, 1267, 1223, 1178, 1020, 920. Anal. Calcd for $C_{18}H_{27}N_2O_5S$: C, 56.38; H, 7.10; N, 7.31; S, 8.36. Found: C, 56.73; H 6.99; N, 6.67; S, 8.13. HRMS EI (m/z) calcd for $C_{18}H_{27}N_2O_5{}^{32}S$. 383.1635, found 383.1638.

2.10. 2,2,5,5-tetramethyl-3-[(2-oxothiolan-3-yl)carbamoyl]-2,5-dihydro-1*H*-pyrrol-1-oxyl (5)

To a stirred solution of 3-carboxy-2,2,5,5-tetramethyl-2,5-dihydro-1*H*-pyrrol-1-oxyl **1** (100 mg, 0.543 mmol) in a mixture of dry chloroform (2 mL) and pyridine (86 mg, 1.09 mmol), a dropwise addition of thionylchloride (70 mg, 0.59 mmol) was made. The mixture was stirred for 20 min at room temperature and after that homocysteine thiolactone hydrochloride (83 mg, 0.543 mmol) was added in one portion to the mixture. Then a solution of triethylamine (130 mg, 1.28 mmol) in dry chloroform (5 mL) was added to the cold stirred mixture. After stirring the mixture was extracted with brine, the organic phase was dried with Na_2SO_4 and the solvent was distilled off under reduced pressure. The residue was separated using column chromatography on silicagel, chloroform to give **5** (109 mg, 70 %): yellow powder, mp 146.1 °C, the subsequent decomposition; UV (EtOH) $\lambda_{max/nm}$ (*lgε*, $M^{-1}cm^{-1}$): 230 (4.1). IR ν_{max} 3361, 3321, 3054, 2976, 2931, 2867, 1706, 1664, 1620, 1531, 1465, 1437, 1356, 1311, 1208, 1161, 1061, 1020, 921, 764, 528. Anal. Calcd for $C_{13}H_{19}N_2O_3S$: C, 55.10; H, 6.76; N, 9.89; S, 11.32. Found: C, 55.22; H 6.54; N, 9.84; S, 11.51. HRMS EI (m/z) calcd for $C_{13}H_{19}O_3N_2{}^{32}S$ 283.1111, found 283.1112

2.11. 2,2,5,5-Tetramethyl-3-[(2-oxothiolan-3-yl)carbamoyl]pyrrolidin-1-oxyl (6)

To a stirred solution of 3-carboxy-2,2,5,5-tetramethylpyrrolidin-1-oxyl **2** (1.0 g, 5.37 mmol) in a mixture of dry chloroform (15 mL) and pyridine (0.862 mg, 10.8 mmol), a dropwise addition of thionyl chloride (0.703 g, 5.9 mmol) was made. The mixture was stirred for 20 min at room temperature and then homocysteine thiolactone hydrochloride (0.825 g, 5,37 mmol) was added in one portion to the mixture. Then a solution of triethylamine (1.3 g, 13 mmol) in dry chloroform (5 mL) was added to the cold stirred mixture. After stirring the mixture was extracted with brine, the organic phase was dried with Na_2SO_4 and the solvent was distilled off under reduced pressure. The residue was separated using column chromatography on silicagel, chloroform to give **6** (1.15 g, 75 %): yellow crystals, mp 174.0 °C, the subsequent decomposition; UV (EtOH) $\lambda_{max/nm}$ (*lgε*, $M^{-1}cm^{-1}$): 235 (3.8). IR ν_{max} 3286, 3066, 2980, 2929, 2908, 1702, 1653, 1523, 1460, 1380, 1363, 1309, 1230, 1172, 1102, 1056, 1022, 918, 858. Anal. Calcd for $C_{13}H_{21}N_2O_3S$: C, 54.71; H, 7.42; N, 9.82; S, 11.84. Found: C, 55.55; H 7.38; N, 10.03; S, 11.56. HRMS EI (m/z) calcd for $C_{13}H_{21}O_3N_2{}^{32}S$ 285.1267, found 285.1269. Crystal data (**6a**) (Fig. S11.1): $C_{13}H_{21}N_2O_3S$, M = 285.38, monoclinic, space group $P2_1/c$, at 296 K: a = 6.5129(5), b = 24.5684(18), c = 9.5983(6) Å, β = 92.042(3)°, V = 1534.86(19) Å^3, Z = 4, d_{calc} = 1.235 g/cm^3, μ = 0.217 mm^{-1}, a total of 30509 I (θ_{max} = 28.01°), 3680 unique (R_{int} = 0.0442), 2860 I > 2σ(I), 180 parameters. GooF = 0.986, R_1 = 0.0608, wR_2 = 0.1583 [I > 2σ(I)], R_1 = 0.0796, wR_2 = 0.1727 (all data), max/min diff. peak 0.387/-0.263 e·Å^{-3}.

2.12. 2,2,5,5-Tetraethyl-3-[(2-oxothiolan-3-yl)carbamoyl]pyrrolidin-1-oxyl (7)

To a stirred solution of 3-carboxy-2,2,5,5-tetraethylpyrrolidin-1-oxyl **3** (0.60 g, 2.48 mmol) in a mixture of dry chloroform (5 mL) and pyridine (400 mg, 5.05 mmol), a dropwise addition of thionyl chloride (0.324 g, 2.72 mmol) was made. The mixture was stirred for 20 min at room temperature and then homocysteine thiolactone hydrochloride (380 mg, 2.48 mmol) was added in one portion to the mixture. Then a solution of triethylamine (610 mg, 6.04 mmol) in dry chloroform (5 mL) was added to a cold stirred mixture. After stirring the mixture was extracted with brine, the organic phase was dried with Na_2SO_4 and the solvent was distilled off under reduced pressure. The residue was separated using column chromatography on silicagel, chloroform to give **7** (505 mg, 60 %): yellow powder, mp 174.0 °C, the subsequent decomposition; UV (EtOH) $\lambda_{max/nm}$ (*lgε*, $M^{-1}cm^{-1}$): 235 (3.77). IR(KBr) ν_{max} 3279, 3068, 2968, 2937, 2881, 1697, 1656, 15446, 1461, 1024, 919. Anal. Calcd for $C_{17}H_{29}O_3N_2S$: C, 59.79; H, 8.56; N, 8.20; S, 9.39. Found: C, 60.06; H, 8.51; N, 8.20; S, 8.20. HRMS EI (m/z) calcd for $C_{17}H_{29}O_3N_2{}^{32}S$ 341.1893, found 341.1895.

3. Synthesis and characterization of HSA-nitroxide conjugates

Synthesis of human serum albumin modified by nitroxide radical homocysteine thiolactone derivatives

The synthetic procedure was adapted from Chubarov *et al.* [10,11] Briefly, a solution of fatty acid free human serum albumin (Sigma A3782, 33.5 mg, 0.53 mM) in PBS (15 mM KH_2PO_4, 145 mM NaCl, pH 7.4 with or without 0.2 mM EDTA) was mixed with 6-60-fold excess of nitroxide radical homocysteine thiolactone (HTL) derivatives (**5**-**8**) in 50 µl DMSO and stirred (500 rpm) at 37 °C for 18 h. The excess of the reagents was removed by filtration using Centricon concentrators (M_r<3 kDa cut off, Amicon Centriprep YM30, Millipore) according to the procedure described by the manufacturer. The amount of nitroxide label per protein molecule was analyzed by Ellman's assay (SH-group reagent), CW EPR and MALDI-ToF (see Fig. S3.1 and Tables S1-S2). For comparison, at the same conditions *N*-homocysteinylated HSA was synthesized using HTL. For the relaxivity, MRI properties etc. studies, conjugates were synthesized using a 30-fold excess of nitroxide radical HTL derivatives (PBS buffer with EDTA).

Albumin has 35 Cys residues, with 34 paired in 17 disulfide bonds. The remaining unpaired Cys34 either is reduced, with a free SH group, (in healthy adults, about 70–80 % of the HSA has this form) or is oxidized as a disulfide (20-25 %) or as a sulfinic or sulfonic acid (2-5 %). The HSA used in this study had 0.29±0.05 sulfhydryl groups per protein molecule.

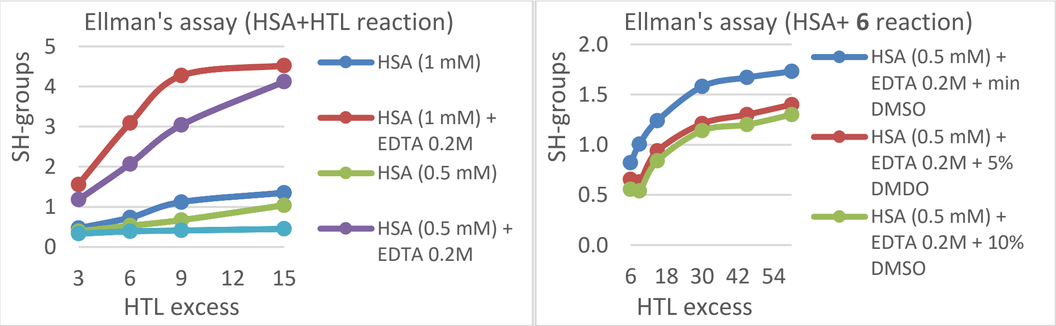

Fig. S3.1. Dependence of SH-group content in albumin conjugate on the HTL or HTL derivative 6 excess

Covalent adduct formation between HTL and protein lysine residues results in the appearence of a new free SH in the modified protein. Hence an increase in the free SH content upon HTL treatment is regarded to be a good signature of protein covalent adduct formation by HTL. Ellman's assay is widely used because of its simplicity and reliable results. Ellman's reagent is converted into its reduced form in the presence of thiols and can be measured directly by UV spectrometry. However, as we can see from Table S1, higher concentrations of albumin and DMSO lead to a lower amount of SH groups. *N*-homocysteinylation makes HSA more susceptible to oxidation, as seen by enhanced disulfide formation that yields disulfide-linked albumin dimers (Fig. S3.2); and by higher oxidation products of its thiol groups, such as sulfinate and sulfonate, at physiological temperature without any other added oxidants [12]. While most oxidizable functional groups are quite stable to DMSO, there are several reports in the literature of its use as a mild oxidizing agent for SH groups [13,14]. Thus, the measured extent of reaction calculated from Ellman's test is much lower than from MALDI ToF (Table S1). Therefore, the synthesized HSA-NIT conjugates were further characterized by EPR and MALDI ToF to obtain the nitroxide content (Table S2).

Table S1. Dependence of modification degree calculated by measuring SH-group content in albumin conjugate and by MALDI TOF data

Reaction condition	Reagent excess	Modification degree calculated by	
		Ellman's test[1]	MALDI TOF
HSA 1 mM	9, HTL	0.8	5.1
HSA 0.5 mM + DMSO 15%	9, HTL	0.2	4.5
HSA 0.5 mM + EDTA (0.2 mM)	6, HTL	1.8	3.5
HSA 0.5 mM + EDTA (0.2 mM) + min DMSO	6, compound **6**	0.8	1.0
	9, compound **6**	1.0	1.3
	15, compound **6**	1.2	2.5
	30, compound **6**	1.6	4.1
	45, compound **6**	1.7	4.5

[1]Amount of SH groups minus 0.29 (SH groups in the native HSA)

Table S2. Modification degree calculated by MALDI TOF and EPR data for HSA-NIT conjugates

HSA type	Modification degree calculated by		
	EPR	MALDI TOF	average
HSA-NIT-5	4.7	4.9	4.8
HSA-NIT-6	3.7	4.1	3.9
HSA-NIT-7	4.1	3.6	3.9
HSA-NIT-8	2.3	2.5	2.4

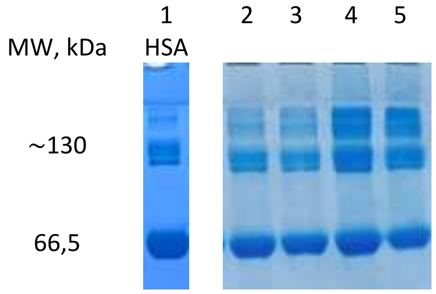

Fig. S3.2. SDS–PAGE of *N*-homocysteinylated albumin under Laemmli condition without addition of DTT with subsequent Coomassie blue staining. HSA (lane 1); Hcy-HSA (HSA 0.5 mM + HTL 6 fold excess, lane 2); Hcy-HSA (HSA 0.5 mM + HTL 9 fold excess, lane 3); Hcy-HSA (HSA 0.5 mM + HTL 6 fold excess + DMSO 15%, lane 4); Hcy-HSA (HSA 0.5 mM + HTL 9 fold excess + DMSO 15%, lane 5)

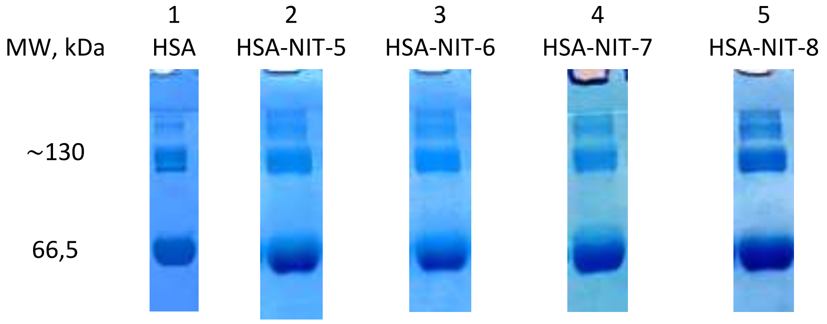

Fig. S3.3. SDS–PAGE of HSA-NIT 5-8 conjugates under Laemmli condition without addition of DTT with subsequent Coomassie blue staining. Quantitative data were obtained by digitizing the gel using GelPro Analyzer software (Media Cybernetics) and presented in the Table 1

Table S3. The parameters for EPR simulations in Fig. 3

Spectra were simulated assuming the presence of a set of fractions characterized by different rotational correlation times (τ_c) and line widths. The g-tensor g_{ii} and hyperfine tensor A_{xx}, A_{yy} values were the same in all spectra: g_{xx}=2.009, g_{yy}=2.006, g_{zz}=2.0025, A_{xx}=A_{yy}=0.5; the values of A_{zz} were varied. Line broadenings were calculated assuming Voigtian line shape – convolution of Lorentzian and Gaussian line shape.

Sample	Component	τ_c, ns	A_{zz}, mT (±0.1 mT)	Weight, %	Gaussian peak-to-peak line width, mT (±0.1 mT)	Lorentzian peak-to-peak line width, mT (±0.1 mT)
HSA-NIT-6	slow	10±5	3.5	70±10	0.1	0.45
	fast	2.1±0.5	3.9	29±10	0.1	0.2
	free	0.6±0.2	3.9	1±0.5	0.1	0
HSA-NIT-5	slow	10±5	3.6	58±10	0.15	0.4
	fast	1.9±0.5	3.9	41±10	0.1	0.55
	free	0.6±0.2	3.8	1±0.5	0.1	0
HSA-NIT-7	slow	14±5	3.5	85±10	0.4	0.85
	fast	2.7±0.5	3.8	15±10	0.1	0.45
	free	0.6±0.2	3.8	0.2±0.1	0.1	0
HSA-NIT-8	slow	14±5	3.5	88±10	0.5	0.2
	fast	2.6±0.5	3.6	12±10	0	0.1
	free	0.6±0.2	3.8	0.2±0.1	0.1	0

4. Identification of *N*-homocysteinylation sites in the HSA-NIT conjugates

In order to identify specific *N*-homocysteinylation sites in HSA-nitroxide conjugates, the modified albumin and HSA were digested with trypsin prior to MS analysis (Promega's protocol for digestion). Prior to analysis, samples were purified and fractionated on ZipTip C18 pipette tips. Purified peptides were eluted directly on a MALDI plate, mixed with α-cyano-4-hydroxy-cinnamic acid (HCCA) (saturated matrix solution dissolved in 50 % acetonitrile 0.1 % TFA) and allowed to air dry. Samples were analyzed in reflectron positive ion mode in the *m/z* range from 500 to 5000 Da; mass calibration was performed using peptide calibration standards (Bruker, Germany). The masses of the peptides detected in the HSA and conjugates digests were compared to masses predicted for a theoretical digest of HSA by the PEPTIDEMASS software (http://web.expasy.org/peptide_mass/). When generating the theoretical digest, the following considerations were made: the maximum number of missed cleavages was 2; the peptide mass range 500–5000 Da was used; all cysteine residues were assumed to be treated with iodoacetamide or without it. Molecular Weight Calculator of Peptides software program from Peptide Protein Research Ltd

(http://www.peptidesynthetics.co.uk/tools/) was used to calculate theoretical digest masses, allowing a mass increase of 283 Da (HSA-NIT-5), 285 Da (HSA-NIT-6), 341 Da (HSA-NIT-7), or 385 Da (HSA-NIT-8) due to *N*-homocysteinylation by the NIT-HTL derivative and of 57 Da due to carbamidomethylation (with IAA). Trypsin does not cut after modified lysine residues, thus *N*-NIT-Hcy-Lys residue cannot be present at the C-terminus. MALDI ToF data was deconvoluted using the software package mMass (http://www.mmass.org). All peptides that could be found in a theoretical or experimental digest of HSA were removed from further analysis.

Sequence coverage was estimated using Mascot software at http://www.matrixscience.com. Direct application of MS for analysis of HSA-NIT conjugates obtained 25-39 % protein sequence coverage (HSA-NIT-5, 29 %; HSA-NIT-6, 39 %; HSA-NIT-7, 34 %; HSA-NIT-8, 25 %), comparable to that of trypsinized *N*-Hcy-albumin samples, whose protein sequence coverage is less than 40 % [15]. For HSA we obtained 67 % protein sequence coverage.

Table S4. Identification of specific *N*-homocysteinylation sites in HSA-NIT conjugates

N-Hcy site	Sequence	peptide m/z calc.	MW calc., peptide MW + …		*m/z* measured	missed cleavages
			+NIT-Hcy	+NIT-Hcy+IAA		
			HSA-NIT-5			
Lys-525	^{525}**_K_***QTALVELVKHKPK^{538}	1,618.0	1,901.1	1,958.1	1,957.7	*3*
		809.0	950.5	979.0	950.3	
	^{522}QIK**_K_***QTALVELVK^{534}	1,496.9	1,780.0	1,837.0	1,780.6	2
		748.5	890.0	918.5	890.5/918.4	
	^{525}**_K_***QTALVELVK^{534}	1,127.7	1,410.8	1,467.8	1,467.7	1
Lys-212	^{210}AF**_K_***AWAVAR^{218}	1,018.6	1,301.7	1,358.7	1,360.5	1
	^{210}AF**_K_***AWAVARLSQR^{222}	1,502.8	1,785.9	1,842.9	1,786.6	2
Lys-205	^{200}CASLQ**_K_***FGERAFK^{212}	1,483.8	1,766.9	1,823.9	1,766.6	2
		741.9	883.4	911.9	883.3/912.4	
	+IAA	1,540.8	1,823.9	1,880.9	-	
	+IAA	770.4	911.9	940.4	912.4/940.3	
	^{200}CASLQ**_K_***FGER^{209}	1,137.5	1,420.7	1,477.7	1,419.5	1
	+IAA	1,194.5	1,477.7	1,534.7	1,534.6	
	^{198}LKCASLQ**_K_***FGER^{209}	1,378.7	1,661.8	1,718.8	1,661.7	2
		689.4	830.9	859.4	859.7	
	+IAA	1,435.7	1718.8	1775.8	-	
	+IAA	717.9	859.4	887.9	889.3	
Lys-159	^{146}HPYFYAPELLFFA**_K_***R^{160}	1,898.0	2,181.1	2,238.1	2,239.8	1
	^{145}RHPYFYAPELLFFA**_K_***R^{160}	2,054.1	2,337.2	2,394.2	-	2
		1,027.0	1,168.6	1,197.1	1,171,3	
Lys-137	^{115}LVRPEVDVMCTAFHDNEETFLK**_K_***Y LYEIAR^{144}	3,628.8	3,911.9	3,968.9	-	3
		1,814.4	1,956.0	1,984.5	1,954.7	
Lys-12	^{11}F**_K_***DLGEENFKALVLIAFAQY LQQCPFEDHVK^{41}	3,639.8	3,922.9	3,979.9	-	2
		1,819.9	1,961.5	1,990.0	-	
	+IAA	3,696.8	3,979.9	4,036.9	-	
	+IAA	1,848.4	1,990.0	2,018.5	2,017.3	
	^{11}F**_K_***DLGEENFK^{20}	1,225.6	1,508.7	1,565.7	1,565.5	1
		612.8	754.3	782.8	756.3	
Lys-4	^{1}DAH**_K_***SEVAHRFK^{12}	1,423.7	1,706.8	1,763.8	1,764.5	2
		711.9	853.4	881.9	881.4	
Lys-560	^{558}CC**_K_***ADDK^{564}	781.3	1,064.3	1,121.3	1,064.4/1,121.2	1
Lys-436	^{433}VGS**_K_***CCK^{439}	723.3	1,006.3	1,063.3	1,064.4	1
Lys-225	^{219}LSQRFP**_K_***AEFAEVSK^{233}	1,735.9	2,018.9	2,075.9	-	2

		868.0	1,009.5	1,038.0	1,039.3	
Lys-162	^{160}RY_**K**_*AAFTECCQAADK^{174}	1,703.8	1,986.8	2,043.8	-	2
		851.9	993.4	1,021.9	1,022.5	
Lys-93	^{82}ETYGEMADCCA_**K**_*QEPER^{98}	1,958.8	2,241.8	2,298.8	-	1
		979.4	1,120.9	1,149.4	1,121.2	
Lys-73	^{65}SLHTLFGD_**K**_*LCTVATLRETYGEMAD CCAK^{93}	3,175.4	3,458.4	3,515.4	-	2
		1,587.7	1,729.2	1,757.7	1,729.0	
Lys-64	^{52}TCVADESAENCD_**K**_*SLHTLFGDK^{73}	2,382.0	2,665.0	2,722.0	-	1
		1,191.0	1,332.5	1,361.0	1,333.6/1,363.4	
	HSA-NIT-6					
Lys-525	^{525}_**K**_*QTALVELVKHKPK^{538}	1,618.0	1,903.1	1,960.1	1,902.8/1,960.0	*3*
		809.0	951.6	980.1	980.4	
	^{522}QIK_**K**_*QTALVELVK^{534}	1,496.9	1,782.0	1,839.0	-	2
		748.5	891.0	919.5	919.5	
Lys-212	^{210}AF_**K**_*AWAVAR^{218}	1,018.6	1,303.7	1,360.7	1,301.5/1,362.6	1
Lys-205	^{200}CASLQ_**K**_*FGERAFK^{212}	1,483.8	1,768.9	1,825.9	1,767.7	2
		741.9	884.4	912.9	912.4	
	+IAA	1,540.8	1,825.9	1,882.9	-	
	+IAA	770.4	912.9	941.4	912.4/942.5	
	^{200}CASLQ_**K**_*FGER^{209}	1,137.5	1,422.7	1,479.7	1,421.0	1
		568.8	711.3	739.8	711.3/739.5	
	+IAA	1194.5	1,479.7	1,536.7	1,536.6	
	+IAA	597.3	739.8	768.3	739.5	
Lys-159	^{146}HPYFYAPELLFFA_**K**_*R^{160}	1,898.0	2,183.1	2,240.1	2,241.8	1
		949.0	1091.6	1120.1	1,121.3	
Lys-137	^{115}LVRPEVDVMCTAFHDNEETFLK_**K**_*Y LYEIAR^{144}	3,628.8	3,913.9	3,970.9	-	3
		1,814.4	1,957.0	1,985.5	1,957.8	
Lys-12	^{11}F_**K**_*DLGEENFKALVLIAFAQYLQQCP FE DHVK^{41}	3,639.8	3,925.0	3,982.0	-	2
		1,819.9	1,962.5	1,991.0	-	
	+IAA	3,696.8	3,982.0	4,039.0	-	
	+IAA	1,848.4	1,991.0	2,019.5	2,019.6	
	^{5}SEVAHRF_**K**_*DLGEENFK^{20}	1,904.9	2,190.1	2,247.1	-	2
		952.5	1,095.0	1,123.5	1,123.4	
Lys-564	^{561}ADD_**K**_*ETCFAEEGK^{573}	1,441.6	1,726.7	1,783.7	1,727.0	1
Lys-351	^{349}LA_**K**_*TYETTLEKCCAAADPHECYAK^{372}	2,658.2	2,943.3	3,000.3	2943.1	2
Lys-444	^{440}HPEA_**K**_*R^{445}	736.4	1,021.5	1,078.5	1,081.0	1
Lys-432	^{429}NLG_**K**_*VGSKCCK^{439}	1,135.6	1,420.7	1,477.7	1,419.9	2
Lys-413	^{411}YT_**K**_*K^{414}	538.3	823.4	880.4	825.0	1
Lys-195	^{191}ASSA_**K**_*QRLK^{199}	987.6	1,272.7	1,329.7	1,330.0	2
Lys-190	^{182}LDELRDEG_**K**_*ASSAK^{195}	1,517.8	1,802.9	1,859.9	1,804.7	2
Lys-73	^{65}SLHTLFGD_**K**_*LCTVATLR^{81}	1,874.0	2,159.1	2,216.1	2,158.9	1
	HSA-NIT-7					
Lys-525	^{522}QIK_**K**_*QTALVELVK^{534}	1,496.9	1838.1	1895.1	1,896.7	2
		748.5	919.1	947.6	949.3	
	^{525}_**K**_*QTALVELVK^{534}	1,127.7	1,468.9	1,525.9	1,468.6	1
		563.8	734.4	762.9	735.1	
Lys-212	^{210}AF_**K**_*AWAVAR^{218}	1,018.6	1,359.8	1,416.8	1,417.8	1
	^{210}AF_**K**_*AWAVARLSQR^{222}	1502.8	1,844.0	1,901.0	1,900.7	2
Lys-205	^{200}CASLQ_**K**_*FGERAFK^{212}	1,483.8	1,824.9	1,881.9		2
		741.9	912.5	941.0	912.3	
	^{200}CASLQ_**K**_*FGER^{209}	1,137.5	1,478.7	1,535.7	-	1
		568.8	739.4	767.9	-	
	+IAA	1194.5	1,535.7	1,592.7	1,535.3/1,593.7	
	+IAA	597.3	767.9	796.4	-	

	^{198}LKCASLQK*FGER^{209}	1,378.7	1,719.9	1,776.9	-	2
		689.4	860.0	888.5	859.8	
	+IAA	1,435.7	1,776.9	1,833.9	-	
	+IAA	717.9	888.5	917.0	917.4	
Lys-159	^{146}HPYFYAPELLFFAK*R^{160}	1,898.0	2,239.2	2,296.2	-	1
		949.0	1,119.6	1,148.1	1,121.3	
	^{146}HPYFYAPELLFFAK*RYK^{162}	2,189.1	2,530.3	2,587.3	-	2
		1,094.6	1,265.2	1,293.7	1,294.5	
Lys-137	^{115}LVRPEVDVMCTAFHDNEETFLKK*Y LYEIAR^{144}	3,628.8	3,970.0	4,027.0	-	3
		1,814.4	1,985.0	2,013.5	1,986.5	
	^{137}K*YLYEIAR^{144}	1,054.6	1,395.8	1,452.8	1,396.5/1,453.8	1
	^{137}K*YLYEIARR^{145}	1,210.7	1,551.9	1,608.9	1,611.4	2
Lys-12	^{11}FK*DLGEENFK^{20}	1,225.6	1,566.8	1,623.8	1,566.5	1
		612.8	783.4	811.9	812.3	
Lys-4	^{1}DAHK*SEVAHRFK^{12}	1,423.7	1,764.9	1,821.9	1,764.6	2
		711.9	882.5	911.0	911.3	
Lys-564/573	^{561}ADDK*ETCFAEEGK*K^{574}	1,569.7	1,910.7	1,967.7	1,970.1	2
Lys-466	^{446}MPCAEDYLSVVLNQLCVLHE K*TPVSDR^{472}	3,058.5	3,399.5	3,456.5	-	1
		1,529.3	1,699.8	1,728.3	1,728.0	
Lys-436/439	^{433}VGSK*CCK*HPEAK^{444}	1,285.6	1,626.6	1,683.6	-	2
		642.8	813.3	841.8	842.3	
Lys-274	^{263}YICENQDSISSK*LK^{276}	1,626.8	1,967.8	2,024.8	1,970.1	1
Lys-225	^{219}LSQRFPK*AEFAEVSK^{233}	1,735.9	2,076.9	2,133.9	-	2
		868.0	1,038.5	1,067.0	1,039.4	
Lys-195	^{191}ASSAK*QRLK^{199}	987.6	1,328.6	1,385.6	1,329.0	2
Lys-190/195	^{187}DEGK*ASSAK*QR^{197}	1,175.6	1,516.6	1,573.6	1,575.1	2
		587.8	758.3	786.8	756.9	
			HSA-NIT-8			
Lys-525	^{525}K*QTALVELVKHKPK^{538}	1,618.0	2,003.2	2,060.2	-	3
		809.0	1,001.6	1,030.1	1,031.2	
	^{522}QIKK*QTALVELVK^{534}	1,496.9	1,882.1	1,939.1	-	2
		748.5	941.0	969.5	971.4	
	^{525}K*QTALVELVK^{534}	1,127.7	1,512,9	1,569.9	-	1
		563.8	756.4	784.9	756.7	
Lys-212	^{210}AFK*AWAVAR^{218}	1,018.6	1,403.7	1,460.7	1,460.7	1
Lys-205	^{200}CASLQK*FGERAFK^{212}	1,483.8	1868,9	1,925.9	-	2
		741.9	934,5	963.0	-	
	+IAA	1,540.8	1,925.9	1,982.9	-	
	+IAA	770.4	963.0	991.5	993.2	
	^{198}LKCASLQK*FGER^{209}	1,378.7	1,763.9	1,820.9	1,764.6	2
		689.4	882.0	910.5	912.4	
	+IAA	1,435.7	1,820.9	1,877.9	-	
	+IAA	717.9	910.5	939.0	912.4	
Lys-159	^{146}HPYFYAPELLFFAK*R^{160}	1,898.0	2,283.2	2,340.2	-	1
		949.0	1,141.6	1,170.1	1,143.2/1,171.3	
Lys-137	^{137}K*YLYEIAR^{144}	1,054.6	1,439.8	1,496.8	1,496.5	1
	^{137}K*YLYEIARR^{145}	1,210.7	1,595.9	1,652.9	-	2
		605.3	797.9	826.4	826.3	
Lys-12	^{11}FK*DLGEENFKALVLIAFAQY LQQCPFEDHVK^{41}	3,639.8	4,025.0	4,082.0	-	3
		1,819.9	2,012.5	2,041.0	-	
	+IAA	3,696.8	4,082.0	4,139.0	-	
	+IAA	1,848.4	2,041.0	2,069.5	2,068.7	
	^{5}SEVAHRFK*DLGEENFK^{20}	1,904.9	2,290.1	2,347.1	-	2
		952.5	1,145.1	1,173.6	1,173.5	
Lys-4	^{1}DAHK*SEVAHR^{10}	1,148.6	1,533.7	1,590.7	1,533.5	1

		574.3	766.9	795.4	768.2/795.3	
	^{1}DAHK*SEVAHRFK^{12}	1,423.7	1,808.9	1,865.9	1,808.5	2
		711.9	904.5	933.0	903.5	
Lys-519	^{501}EFNAETFTFHADICTLSEK*ER^{521}	2,487.1	2,872.1	2,929.1	-	
		1,243.5	1,436.05	1,464.55	1,465.4	1
Lys-475	^{473}VTK*CCTESLVNRRPCFSALEVDETY VPK^{500}	3,186.5	3,571.5	3,628.5	-	2
		1,593.3	1,785.8	1,814.3	1,786.0	
Lys-444	^{440}HPEAK*R^{445}	736.4	1,121.4	1,178.4	1,121.0	1
Lys-413	^{411}YTK*K^{414}	538.3	923.3	980.3	922.4/982.0	1
Lys-317/323	^{314}DVCK*NYAEAK*DVFLGMFLYEYA R^{336}	2,744.3	3,129.3	3,186.3	-	2
		1,372.2	1,564.7	1,593.2	1,565.5	
Lys-274	^{263}YICENQDSISSK*LK^{276}	1,626.8	2,011.8	2,068.8	-	1
		813.4	1,005.9	1,034.4	1,035.3	
Lys-199	^{198}LK*CASLQK^{205}	889.5	1,274.5	1,331.5	1,332.5	1
Lys-181	^{175}AACLLPK*LDELRDEGK^{190}	1,769.9	2154,9	2211,9	-	2
			1,077.5	1,106.0	1,079.0	
Lys-162	^{160}RYK*AAFTECCQAADK^{174}	1,703.8	2,088.8	2,145.8	2,089.7	2

5. The pK_a and accessible surface area (ASA) of lysine residues

ASA coefficients was predicted using the VADAR web server (http://redpoll.pharmacy.ualberta.ca/vadar) [16]. Parameters: PDB (1A06, crystal structure of HSA), *Shrake* calculation mode. pK_a values of lysine residues were calculated by PROPKA web server, Propka 3.0 software, and were assigned at pH 7.40 (http://nbcr-222.ucsd.edu/pdb2pqr_2.0.0/ or http://www.propka.org/) [17,18].

Table S5. Computer modelling (pK_a, ASA) of usually *N*-homocysteinylated Lys residues in HSA

HSA 1AO6[a]		
Lys	pK_a	ASA
**4**	-	-
**12**	10.58	0.58
**137**	10.51	0.43
**159**	10.81	0.37
**205**	10.50	0.63
**212**	10.67	0.33
**525**	9.98	0.06

The pK_a and ASA of the *N*-terminus (Lys-4) couldn't be calculated because this portion of HSA doesn't have a well-defined crystal structure. [a]PDB id. code.

Table S6. Computer modelling (pK_a, ASA) of all Lys residues in HSA

HSA 1AO6[a]

Lys	pK_a	ASA	Lys	pK_a	ASA
**12**	10.58	0.58	**323**	10.62	0.45
**20**	10.51	0.26	**351**	10.86	0.51
**41**	11.15	0.46	**359**	10.41	0.69
**51**	10.74	0.42	**372**	10.62	0.59
**64**	10.45	0.3	**378**	10.7	0.58
**73**	10.82	0.22	**389**	10.46	0.57
**93**	10.36	0.43	**402**	10.45	0.51
**106**	7.36	0.09	**413**	9.49	0.19
**136**	9.68	0.22	**414**	8.47	0.06
**137**	10.51	0.43	**432**	9.23	0.29
**159**	10.81	0.37	**436**	9.5	0.45
**162**	10.58	0.36	**439**	10.49	0.74
**174**	11.13	0.29	**444**	10.27	0.48
**181**	9.98	0.31	**466**	10.88	0.46
**190**	8.67	0.36	**475**	10.39	0.51
**195**	9.99	0.42	**500**	10.43	0.71

**199**	6.78	0.13	**519**	10.33	0.59
**205**	10.5	0.63	**524**	10.24	0.45
**212**	10.67	0.33	**525**	9.98	0.06
**225**	10.94	0.46	**534**	9.16	0.1
**233**	11.17	0.35	**536**	10.5	0.2
**240**	10.93	0.52	**538**	10.51	0.93
**262**	10.83	0.54	**541**	10.43	0.78
**274**	11.09	0.24	**545**	10.1	0.46
**276**	10.72	0.55	**557**	10.46	0.57
**281**	10.39	0.44	**560**	10.34	0.57
**286**	10.09	0.17	**564**	10.51	0.82
**313**	10.13	0.73	**573**	10.42	0.61
**317**	11.45	0.66	**574**	10.48	0.7

The pK_a and ASA of the *N*-terminus (Lys-4) couldn't be calculated because this portion of HSA doesn't have a well-defined crystal structure. [a]PDB id. code.

6. Reduction of HSA-NIT conjugates

The reduction condition was adapted from Dobrynin *et al* [84]. The reduction rate constants were investigated using two methods: EPR and T_1-relaxation time measurements [29]. For a typical experiment, stock solutions of ascorbic acid (1 M) and glutathione (GSH, 0.5 M) in PBS buffer pH 7.4 were prepared.

***T_1-relaxation time measurements*.** A portion (0.35 ml) of 0.1 mM HSA-nitroxide conjugate solution in PBS was placed into an NMR tube, and constant nitrogen flow passed through the solution to provide oxygen removal and mixing. The T_1-relaxation time of water in the presence of the HSA-nitroxide conjugates was measured as described below. Ascorbic acid and GSH in 15 µl aliquots was added to the albumin solution. The NMR tube was immediately placed to the NMR spectrometer and the $1/T_1$-relaxation rates were measured as a function of time. The nitroxide concentration was calculated from equation S6.1. The reduction constants were calculated as a pseudo first order rate from the dependence of the logarithm of the difference of the relaxation rates ($1/T_{1,obs}$ and $1/T_{1,d}$) versus time, divided by the concentration of ascorbic acid (C_{asc} – 0.0395 M) (see Eq. S6.3, Fig. S6.1).

$$\frac{1}{T_{i,obs}} = \frac{1}{T_{i,d}} + r_i \cdot C_{NIT}$$

Eq. S6.1

$$C_{NIT} = C_{NIT}^0 \cdot e^{-k \cdot C_{asc} \cdot t}$$

Eq. S6.2

$$\ln\left(\frac{1}{T_{1,obs}} - \frac{1}{T_{1,d}}\right) = \ln(r_1 \cdot C_{NIT}^0) - k \cdot C_{asc} \cdot t$$

Eq. S6.3

Table S7. T_1-relaxation time measurements under reducing conditions for HSA-NIT conjugates

HSA-NIT-5		HSA-NIT-6		HSA-NIT-7		HSA-NIT-8	
Time, min	T_1, msec	Time, min	T_1, msec	Time, min	T_1, msec	Time, min	T_1, msec
0	1958	0	1957	0	2239	0	2647
34.5	3038	34.5	2850	34.5	2395	34.5	2719
67	3169	70	3037	67	2549	67	2836
99.5	3256	105.5	3122	99.5	2648	99.5	2908
		141	3183	132	2713	132	2931
				164.5	2776	164.5	3008
				197	2836	197	3045
				229.5	2864		
				262	2924		
				294.5	2973		
				327	3028		
				359.5	3042		
				392	3057		
				424.5	3106		
				457	3113		
				489.5	3123		

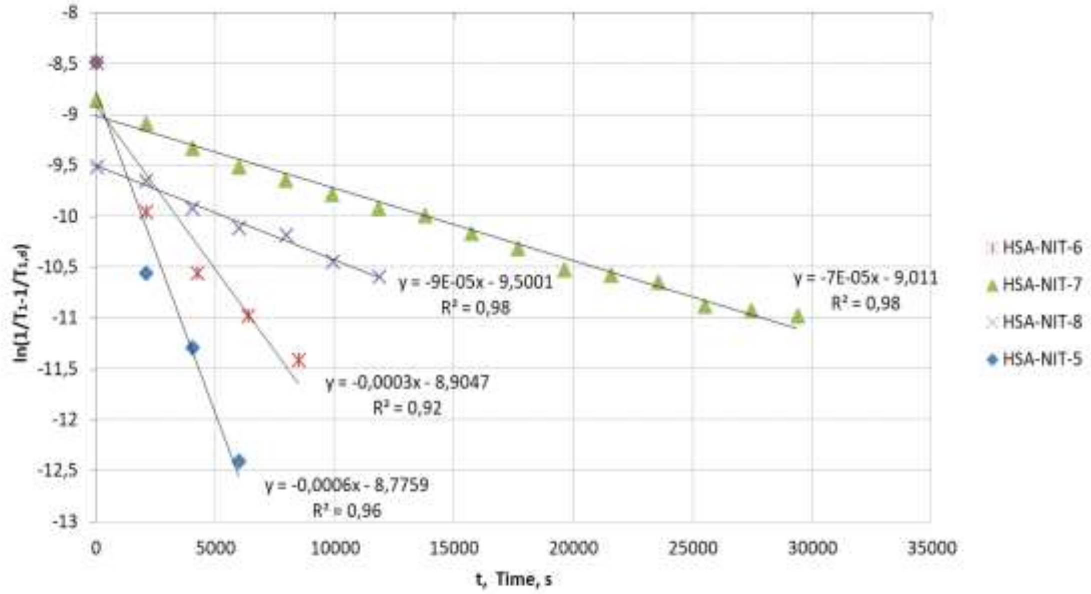

Fig. S6.1. Kinetics of the reduction of HSA-NIT by ascorbate (0.0395 M)/glutathione solution in pH 7.4 at 25 °C by T_1-mes.

EPR. The same necessary aliquots of ascorbic acid, GSH, and HSA-nitroxide conjugate were added to the same buffer solution in consecutive order, and 10 µl of the final solution was drawn into a capillary tube, sealed from both sides, and placed into the resonator of the spectrometer. The double integral of the spectrum was measured as a function of time. CW EPR settings for reduction experiments with HSA-NIT-7 and HSA-NIT-6 were as stated above, except conversion time 20.48 ms, microwave power 6.315 mW, microwave frequency 9.86 GHz, number of scans 1. For HSA-NIT-5 CW EPR settings were: central field 351.6 mT, sweep width 10 mT, microwave power 6.315 mW, microwave frequency 9.87 GHz, modulation frequency 100 kHz, modulation amplitude 0.15 mT, conversion time 81.92 ms, number of points 1024, number of scans 2. For HSA-NIT-8 CW EPR settings were: central field 351.7 mT, sweep width 10 mT, microwave power 6.315 mW, microwave frequency 9.86 GHz, modulation frequency 100 kHz, modulation amplitude 0.2 mT, conversion time 20.48 ms, number of points 1024, number of scans 1. The reduction constant was calculated as a pseudo first order rate equation ($\ln C_{NIT} = \ln C^{o}_{NIT} - kC_{ascorbic\ acid}t$).

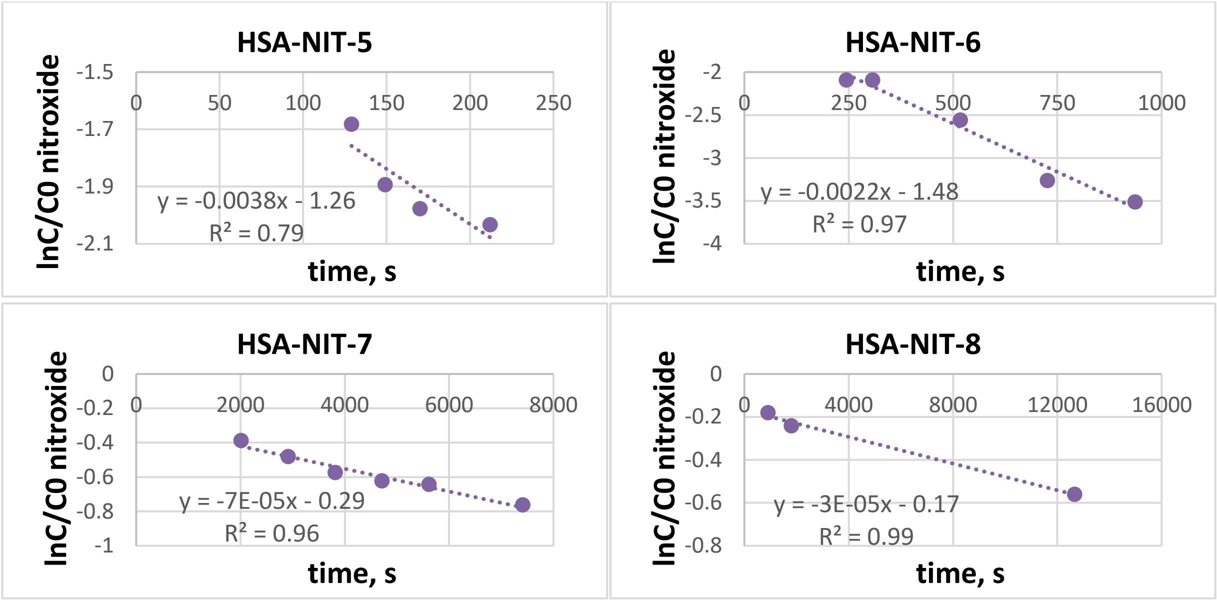

Fig. S6.2. Kinetics of the reduction of HSA-NIT by ascorbate (0.0395 M)/glutathione solution in pH 7.4 at 25 °C by EPR

7. Relaxivity (r_1 and r_2) of HSA-NIT conjugates

Relaxivities of the HSA-nitroxide conjugates were measured at 25 °C (or 37 °C) and 300 MHz (7.0 T) using a Bruker Avance III 300 MHz spectrometer and aqueous solutions buffered at pH 7.4 (PBS) containing 10 % external D_2O for locking purposes. Inversion recovery (T_1) and Car-Purcell-Meiboom-Gill (T_2) pulse sequences was used. The longitudinal and transverse relaxivities, r_1 and r_2, were calculated from equation $1/T_{i,obs} = 1/T_{i,d} + r_i \times [HSA]$, where $T_{i,obs}$ is the measured T_1 (i = 1) or T_2 (i = 2), T_{id} is the diamagnetic contribution of the solvent and [HSA] is the actual HSA-nitroxide conjugate concentration. All reported relaxivity values were the average of three or more independent measurements. $C_{HSA\text{-}NIT}$= 0 mM, $r_{1\ 25°C}$ = 0.35 s^{-1}; $r_{1\ 37°C}$ = 0.30 s^{-1}; $r_{2\ 25°C}$ = 0.71 s^{-1}; $r_{2\ 37°C}$ = 0.60 s^{-1}.

***T*₁-relaxation times at different nitroxide concentrations**	***T*₂-relaxation times at different nitroxide concentrations**

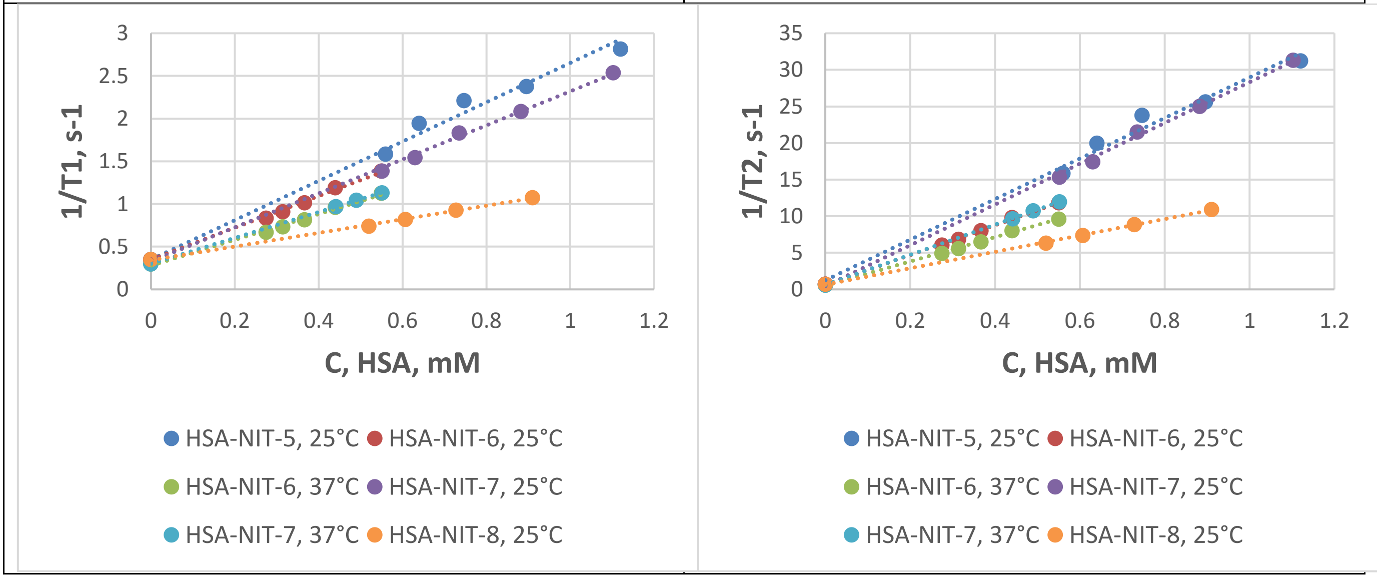

Fig. S7.1. The water proton relaxation times in PBS solution pH 7.4 at 25 °C or 37 °C 7 T magnetic field (300 MHz ^{1}H-frequency)

8. Cell culture and toxicity assay

Tumor cell lines from human mammary adenocarcinoma MCF-7 and human glioblastoma T98G were cultured in IMDM and EMEM medium, respectively, supplemented with 10 % fetal bovine serum (FBS) (Invitrogen), penicillin (100 units/mL), and streptomycin (100 µg/mL) at 37 °C, 5 % CO_2 in humid atmosphere.

The inhibition of cell proliferation was determined using a colorimetric assay based on the cleavage of MTT (3-(4,5-dimethylthiazol-2-yl)-2,5-diphenyltetrazolium bromide) by mitochondrial dehydrogenases in viable cells, leading to formazan formation.[19] Briefly, exponentially growing cells were plated in a 96-well plate (2000 cells per well). After overnight incubation, the cells were treated with media containing albumin or albumin conjugates. The solutions of conjugates were applied in media for 72 h at 37 °C. A 10 µL aliquot of MTT solution (25 mg/mL in PBS) was added to each well, and the plates were incubated at 37 °C for 3 h. The medium was removed, and the dark blue crystals of formazan were dissolved in 0.1 mL of isopropanol. The absorbance at 570 nm (peak) and 620 nm (baseline) was read using a microplate reader Multiscan EX (Thermo Electron Corporation). Results were expressed as a percentage of the control values. All values in the present study are given as the mean ± standard deviation (SD) values, and all measurements were repeated three times.

9. Infrared spectra

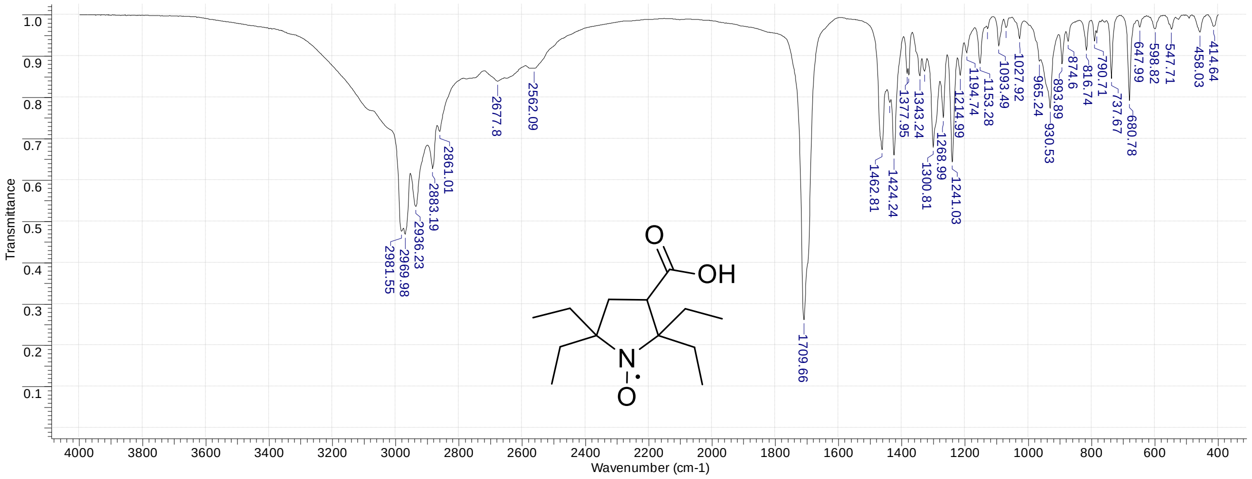

Fig. S9.1. IR(KBr) - 3-Carboxy-2,2,5,5-tetraethylpyrrolidin-1-oxyl (1)

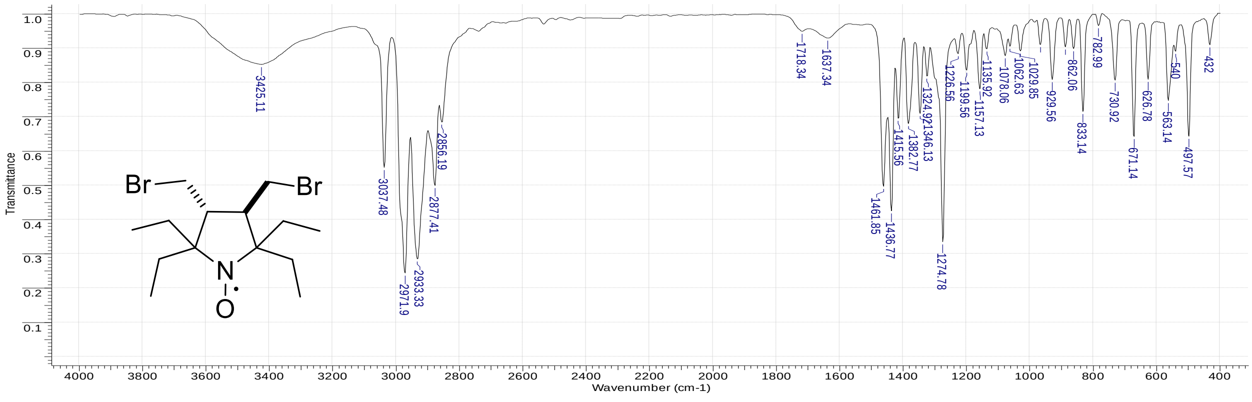

Fig. S9.2. IR(KBr) - 3,4-Bis-(bromomethyl)-2,2,5,5-tetraethylpyrrolidin-1-oxyl (11)

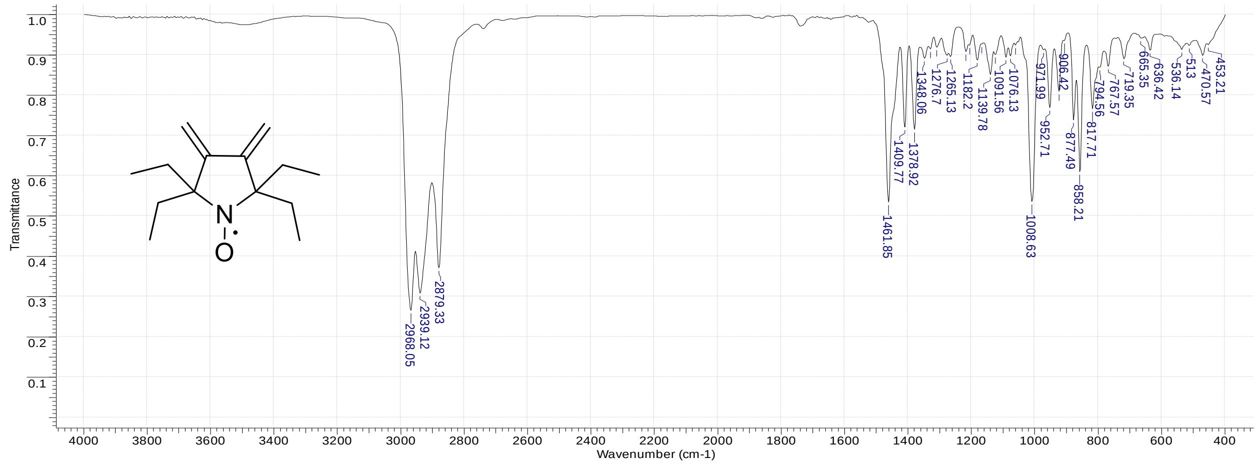

Fig. S9.3. IR(neat) - 2,2,5,5-Tetraethyl-3,4-dimethylidenepyrrolidin-1-oxyl (12)

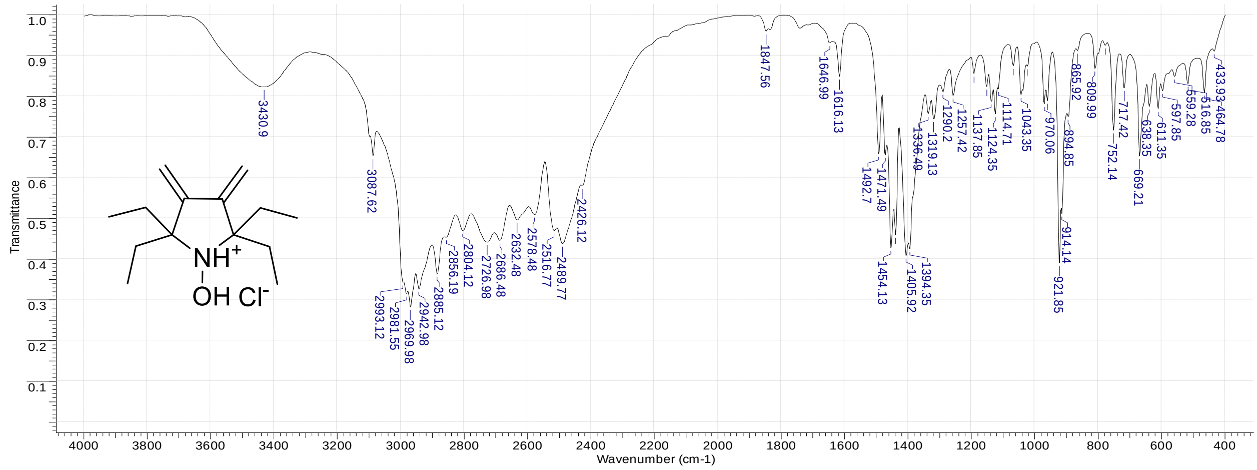

Fig. S9.4. IR (KBr) - 1-Hydroxy-3,4-dimethylidene-2,2,5,5-tetraethylpyrrolidin hydrochloride (13)

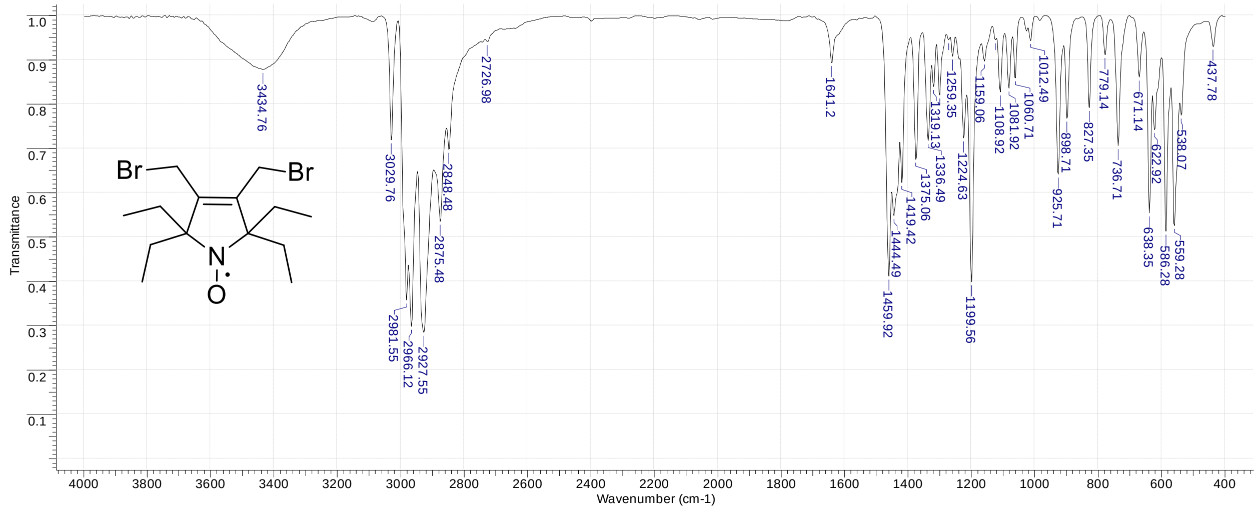

Fig. S9.5. IR (KBr) - 3,4-Bis(bromomethyl)-2,2,5,5-tetraethyl-2,5-dihydro-1*H*-pyrrol-1-oxyl (14)

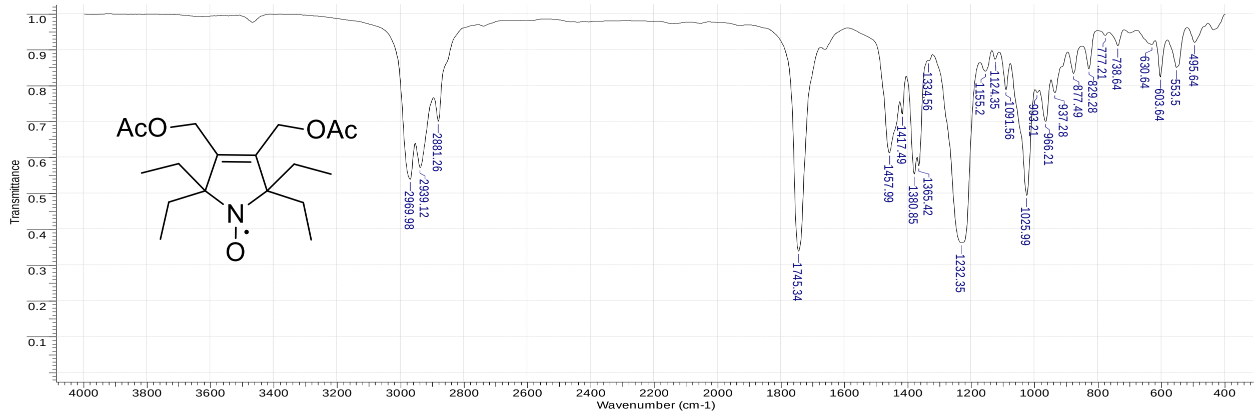

Fig. S9.6. IR (KBr) - 3,4-Bis-[(acetyloxy)methyl]-2,2,5,5-tetraethyl-2,5-dihydro-1*H*-pyrrol-1-oxyl (15)

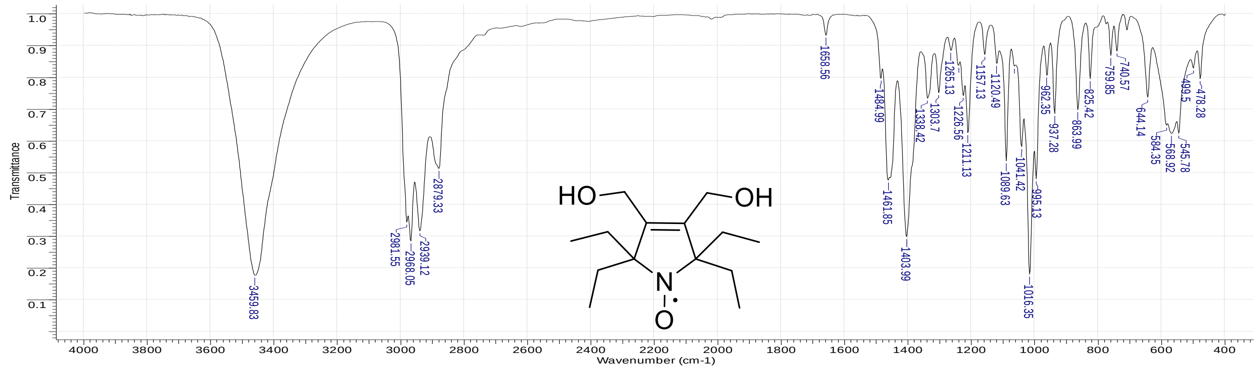

Fig. S9.7. IR(KBr) 2,2,5,5-Tetraethyl-3,4-bis(hydroxymethyl)-2,5-dihydro-1*H*-pyrrol-1-oxyl (16)

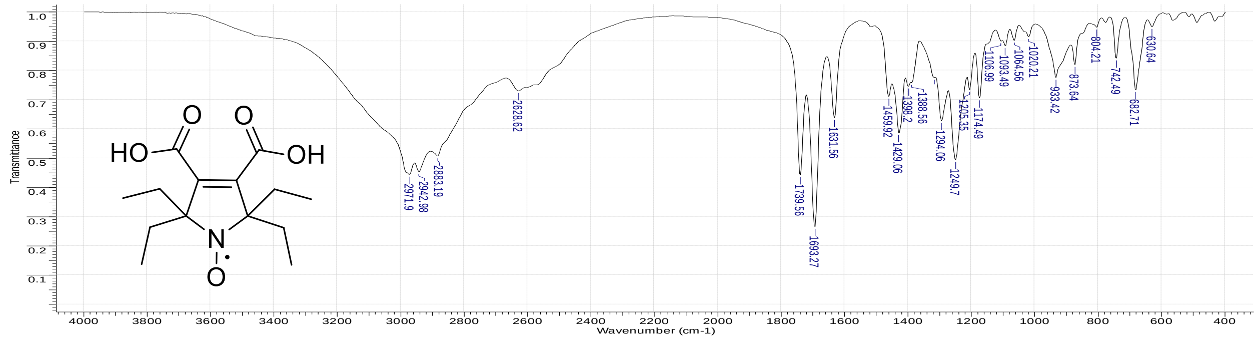

Fig. S9.8. IR (KBr) - 3,4-Dicarboxy-2,2,5,5-tetraethyl-2,5-dihydro-1*H*-pyrrol-1-oxyl (4)

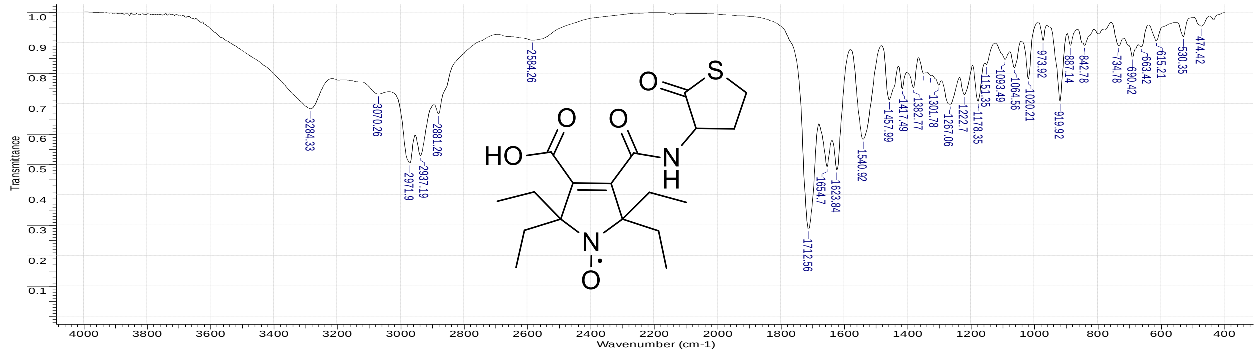

Fig. S9.9. IR (KBr) - 3-Carboxy-2,2,5,5-tetraethyl-4-[(2-oxothiolan-3-yl)carbamoyl]-2,5-dihydro-1*H*-pyrrol-1-oxyl (8)

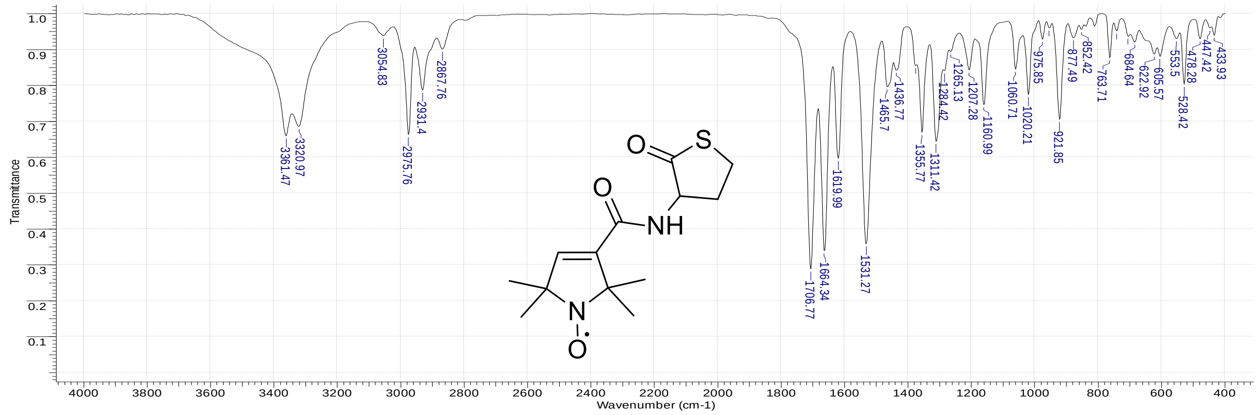

Fig. S9.10. IR (KBr) – 2,2,5,5-tetramethyl-3-[(2-oxothiolan-3-yl)carbamoyl]-2,5-dihydro-1*H*-pyrrol-1-oxyl (5)

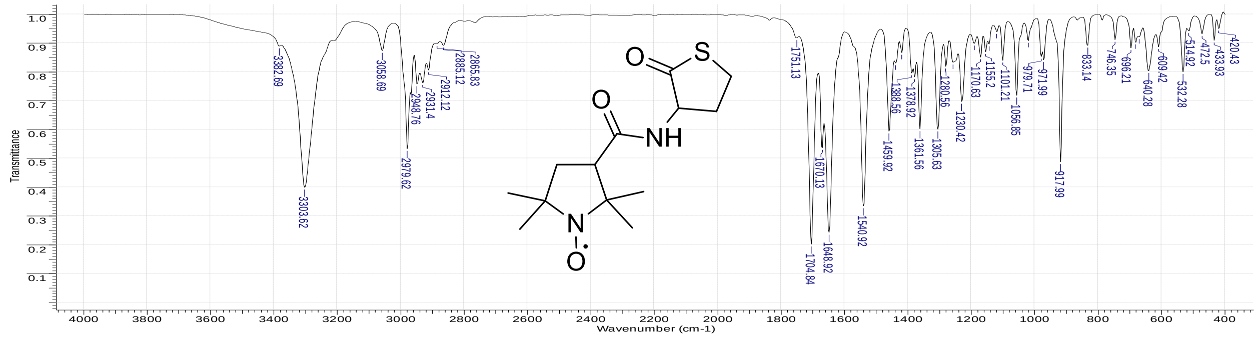

Fig. S9.11. IR (KBr) - 2,2,5,5-Tetramethyl-3-[(2-oxothiolan-3-yl)carbamoyl]pyrrolidin-1-oxyl (6a)

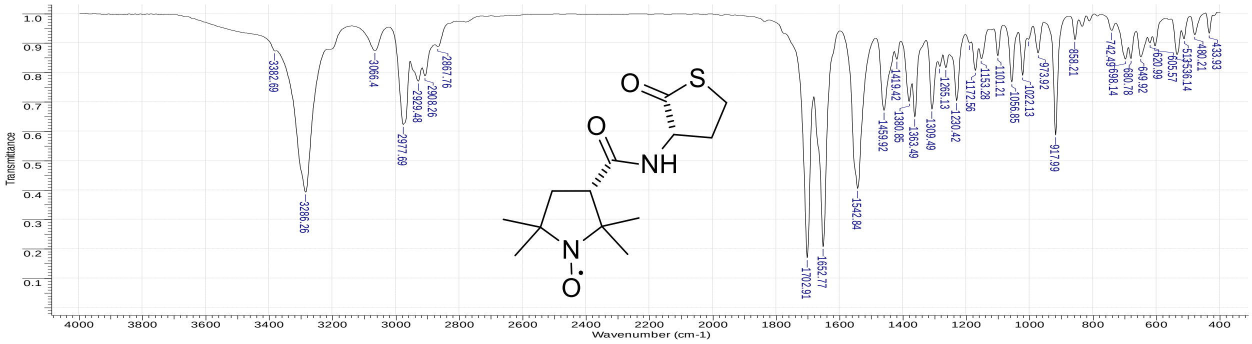

Fig. S9.12. IR (KBr) - 2,2,5,5-Tetramethyl-3-[(2-oxothiolan-3-yl)carbamoyl]pyrrolidin-1-oxyl (6b)

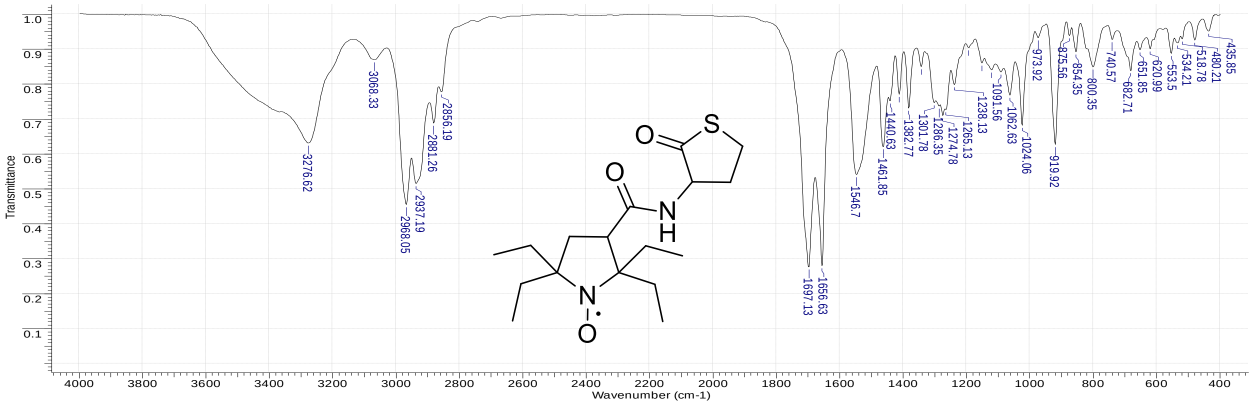

Fig. S9.13. IR (KBr) - 2,2,5,5-Tetraethyl-3-[(2-oxothiolan-3-yl)carbamoyl]pyrrolidin-1-oxyl (7). Mixture of isomers

10.NMR spectra

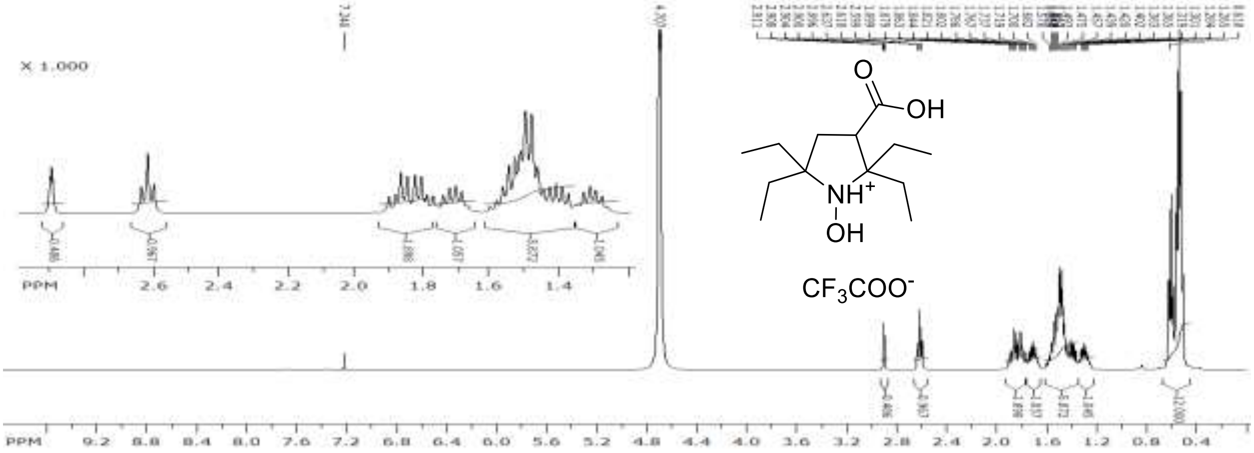

Fig. S10.1. ^{1}H NMR - 3-carboxy-2,2,5,5-tetraethyl-1-hydroxypyrrolidin-1-ium 2,2,2-trifluoroacetate

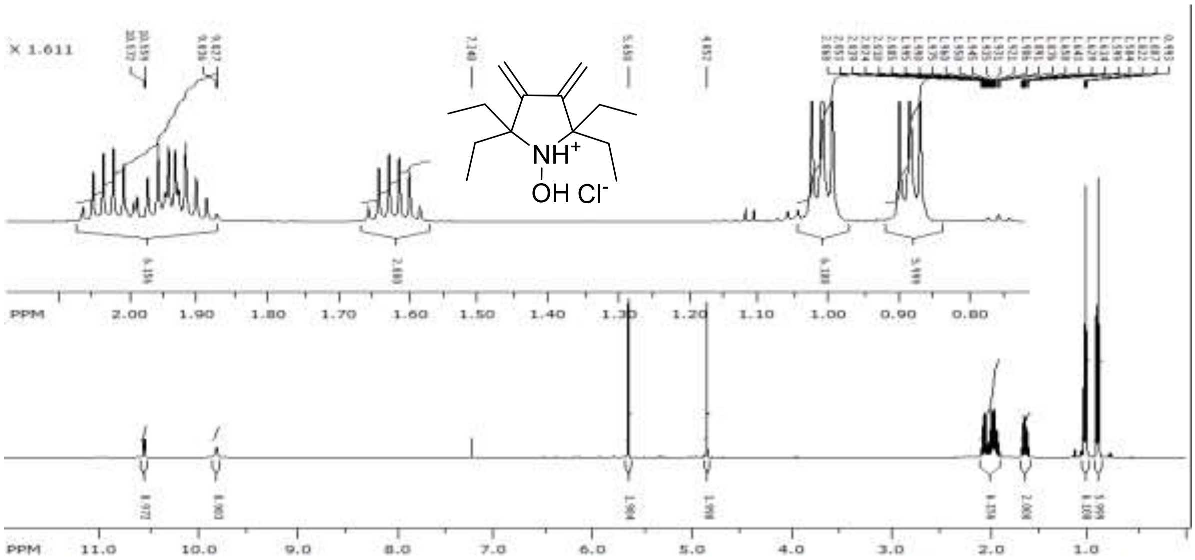

Fig. S10.2. ^{1}H NMR - 1-Hydroxy-3,4-dimethylidene-2,2,5,5-tetraethylpyrrolidin hydrochloride (13)

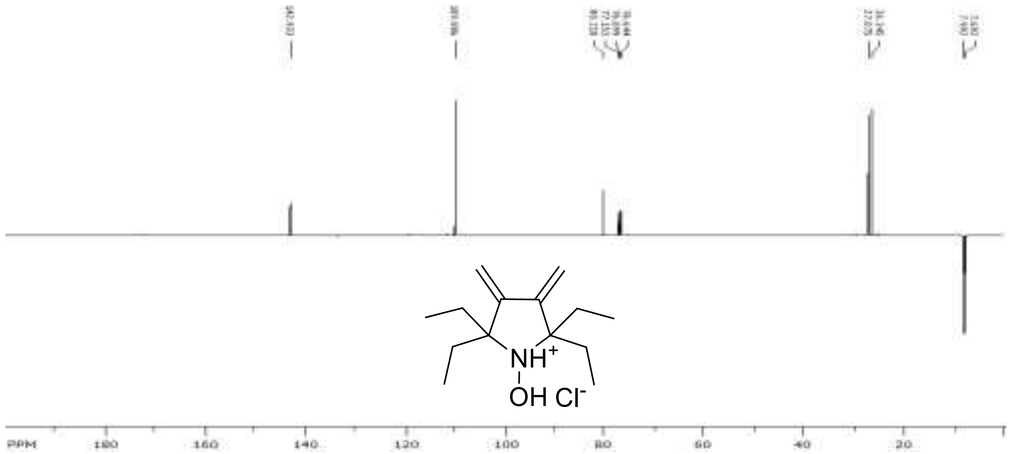

Fig. S10.3. **^{13}C {^{1}H} NMR - 1-Hydroxy-3,4-dimethylidene-2,2,5,5-tetraethylpyrrolidin hydrochloride (13)**

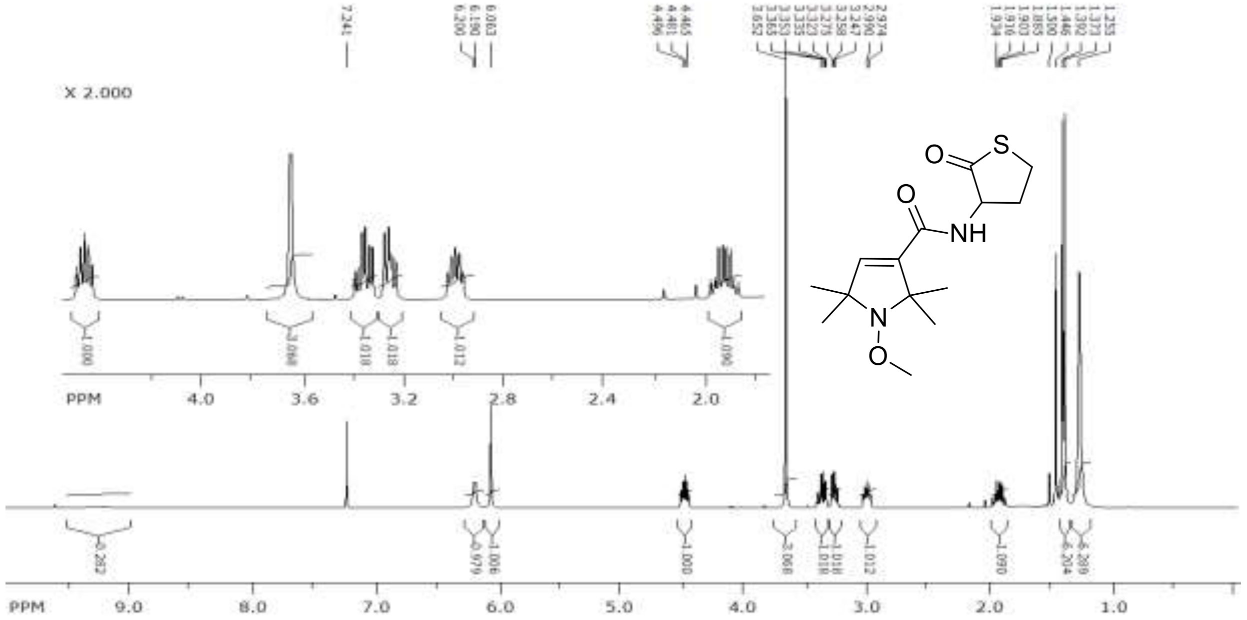

Fig. S10.4. **^{1}H NMR 1-methoxy-2,2,5,5-tetramethyl-3-[(2-oxothiolan-3-yl)carbamoyl]-2,5-dihydro-1*H*-pyrrol**

11. X-ray data for 6a, 13 and 16 compounds

X-ray crystallographic data were obtained on a Bruker Kappa Apex II diffractometer with CCD using graphite-monochromated MoK radiation (λ = 0.71073 Å). Experimental data reduction was performed using APEX2 suite (Bruker AXS Inc. APEX2 (Version 2.0), SAINT (Version 8.18c), and SADABS (Version 2.11); Bruker Advanced X-ray Solutions, Madison, WI, USA, 2000–2012). The structures were solved by direct methods and refined by the full-matrix least-squares technique against F^2 in the anisotropic-isotropic approximation. The H atoms positions were calculated with the riding model. All calculations were performed using SHELXTL-2018/3 [20]. CCDC 1972944 (**6a**), 1972945 (**13**), 1972946 (**16**) contain the supplementary crystallographic data for this paper. These data can be obtained free of charge from The Cambridge Crystallographic Data Centre via http://www.ccdc.cam.ac.uk/conts/retrieving.html (or from the CCDC, 12 Union Road, CambridgeCB2 1EZ, UK; Fax: +44-1223-336033; E-mail: deposit@ccdc.cam.ac.uk).

Chemical structures of the molecules **6a**, **13** and **16** obtained by X-ray single crystal diffractometry.

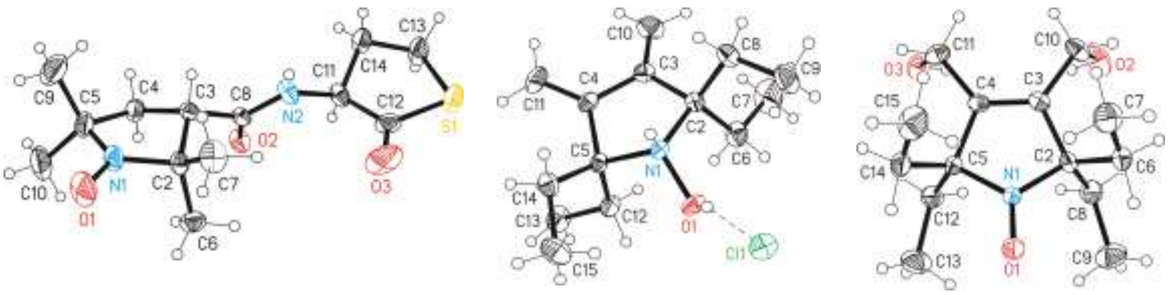

6a **13** **16**

Fig. S11.1. Crystal structure for compounds 6a, 13 and 16 (30% probability ellipsoids)

References

1. Dobrynin, S.A. Khoroshunova, Y.V.; Kirilyuk, I.A. Synthesis of 2,2,5,5-tetraethyl-3-carboxypyrrolidine 1-oxyl. 2019, RU. Patent No. 2 702 331.
2. Dichtl, A.; Seyfried, M.; Schoening, K.U. A novel method for the synthesis of N-alkoxyamines starting from nitroxide radicals and ketones. *Synlett.* **2008**, *2008*, 1877–1881.
3. Paletta, J.T.; Pink, M.; Foley, B.; Rajca, S.; Rajca, A. Synthesis and reduction kinetics of sterically shielded pyrrolidine nitroxides. *Org. Lett.* **2012**, *14*, 5322–5325.
4. Dobrynin, S.A.; Glazachev, Y.I.; Gatilov, Y. V.; Chernyak, E.I.; Salnikov, G.E.; Kirilyuk, I.A. Synthesis of 3,4-Bis(hydroxymethyl)-2,2,5,5-tetraethylpyrrolidin-1-oxyl via 1,3-Dipolar Cycloaddition of Azomethine Ylide to Activated Alkene. *J. Org. Chem.* **2018**, *83*, 5392–5397.
5. Rajca, A.; Wang, Y.; Boska, M.; Paletta, J.T.; Olankitwanit, A.; Swanson, M.A.; Mitchell, D.G.; Eaton, S.S.; Eaton, G.R.; Rajca, S. Organic Radical Contrast Agents for Magnetic Resonance Imaging. *J. Am. Chem. Soc.* **2012**, *134*, 15724–15727.
6. Stoll, S.; Schweiger, A. Easyspin: simulating CW ESR spectra. *Biol. Magn. Reson.* **2007**, *27*, 299–321.
7. Stoll, S.; Schweiger, A. EasySpin, a comprehensive software package for spectral simulation and analysis in EPR. *J. Magn. Reson.* **2006**, *178*, 42–55.
8. Perczel, A.; Hollósi, M.; Tusnády, G.; Fasman, G.D. Convex constraint analysis: a natural deconvolution of circular dichroism curves of proteins. *Protein Eng.* **1991**, *4*, 669–679.
9. Janatova, J.; Fuller, J.K.; Hunter, M.J. The heterogeneity of bovine albumin with respect to sulfhydryl and dimer content. *J. Biol. Chem.* **1968**, *243*, 3612–3622.
10. Chubarov, A.S.; Zakharova, O.D.; Koval, O.A.; Romaschenko, A.V.; Akulov, A.E.; Zavjalov, E.L.; Razumov, I.A.; Koptyug, I.V.; Knorre, D.G.; Godovikova, T.S. Design of protein homocystamides with enhanced tumor uptake properties for 19F magnetic resonance imaging. *Bioorg. Med. Chem.* **2015**, *23*, 6943–6954.
11. Chubarov, A.S.; Shakirov, M.M.; Koptyug, I.V.; Sagdeev, R.Z.; Knorre, D.G.; Godovikova, T.S. Synthesis and characterization of fluorinated homocysteine derivatives as potential molecular probes for 19F magnetic resonance spectroscopy and imaging. *Bioorg. Med. Chem. Lett.* **2011**, *21*, 4050–4053.
12. Sibrian-Vazquez, M.; Escobedo, J.O.; Lim, S.; Samoei, G.K.; Strongin, R.M. Homocystamides promote free-radical and oxidative damage to proteins. *Proc. Natl. Acad. Sci. U. S. A.* **2010**, *107*, 551–554.
13. Yiannios, C.N.; Karabinos, J.V. Oxidation of Thiols by Dimethyl Sulfoxide. *J. Org. Chem.* **1963**, *28*, 3246–3248.
14. Papanyan, Z.K. Interaction of L-cysteine with dimethyl sulfoxide in mild conditions. *Proc. Yerevan State Univ.* **2013**, *2*, 11–14.
15. Marczak, L.; Sikora, M.; Stobiecki, M.; Jakubowski, H. Analysis of site-specific N-homocysteinylation of human serum albumin in vitro and in vivo using MALDI-ToF and LC-MS/MS mass spectrometry. *J. Proteomics* **2011**, *74*, 967–974.
16. Willard, L.; Ranjan, A.; Zhang, H.; Monzavi, H.; Boyko, R.F.; Sykes, B.D.; Wishart, D.S. VADAR: A web server for quantitative evaluation of protein structure quality. *Nucleic Acids Res.* **2003**, *31*, 3316–3319.
17. Olsson, M.H.M.; Søndergaard, C.R.; Rostkowski, M.; Jensen, J.H. PROPKA3: Consistent Treatment of Internal and Surface Residues in Empirical pKa Predictions. *J. Chem. Theory Comput.* **2011**, *7*, 525–537.
18. Søndergaard, C.R.; Olsson, M.H.M.; Rostkowski, M.; Jensen, J.H. Improved treatment of ligands and coupling effects in empirical calculation and rationalization of pKa values. *J. Chem. Theory Comput.* **2011**, *7*, 2284–2295.
19. Mosmann, T. Rapid colorimetric assay for cellular growth and survival: application to proliferation and cytotoxicity assays. *J. Immunol. Methods* **1983**, *65*, 55–63.
20. Sheldrick, G.M. Crystal structure refinement with SHELXL. *Acta Cryst.* **2015**, *C71*, 3–8.
